# Change in the demographic dynamics of a spatially structured population of a colonial waterbird after a perturbation affecting a large colony

**DOI:** 10.1098/rspb.2026.0388

**Published:** 2026-10-14

**Authors:** Killian A. Gregory, Odin Rumianowski, Jean-Dominique Lebreton, Pierre-André Crochet, Aurelien Besnard

**Affiliations:** ^1^ CEFE, Univ Montpellier, CNRS, EPHE, IRD, Montpellier, France; ^2^ CEFE, Univ Montpellier, CNRS, EPHE-PSL University, IRD, Montpellier, France

**Keywords:** black-headed gull, *Chroicocephalus ridibundus*, colonies, conspecific attraction, demography, dispersal, disturbance, larids, patch occupancy, spatially structured population

## Abstract

Localized perturbations may affect spatially structured populations (SSPs) as a whole by altering dispersal between patches. Yet, such large-scale consequences remain poorly understood and may depend on the proportion of the SSP affected by the perturbation. Here, we used correlative analyses and integrated population models to investigate the impact of pond draining—localized perturbations of breeding site availability—on the structure and demographics of an SSP of black-headed gulls (*Chroicocephalus ridibundus*). Pond draining regularly affected patches occupied by colonies, reducing the occupancy probability of those patches in subsequent years, but causing no visible changes in population growth or structure as breeders relocated to unperturbed patches. However, the draining of the pond occupied by the largest colony in the system (64% of total breeders) triggered the immediate emigration of over 60% of breeders outside the SSP, with no subsequent recovery. In the following years, apparent survival showed increased interannual variability, suggesting greater sensitivity of the SSP to the perturbation of smaller colonies. Dispersal between colonies also became more uniform, reflecting the homogenization of colony sizes after the emigration event in a context of informed dispersal. Considering perturbation magnitude and dispersal mechanisms appears essential for understanding the response and resilience of SSPs to local perturbations.

## 1. Introduction

Perturbations are sudden and extreme changes in environmental conditions that affect ecological dynamics [1]). They result from natural (e.g. [2]) or anthropogenic factors (e.g. [3]) and can be one-time events (e.g. [4]), occur repeatedly (e.g.[5]) or durably affect the environment (e.g. [6]). In a spatially structured population (SSP) where individuals occupy patches connected by dispersal [7], a localized perturbation affects only part of the population. Yet, because patch dynamics depend on the dynamics of the other patches to which they are connected [8], perturbation effects inevitably propagate and interfere with SSP functioning. Consequently, predicting the effects of perturbations on SSPs remains challenging [1].

One of the most common behavioural responses to perturbations is the dispersal of individuals from perturbed patches [4,5,9,10], allowing them to mitigate perturbation effects by emigrating from the SSP or relocating to other patches [11–13]. These relocations rapidly propagate local perturbation effects within the SSP. They can promote the colonization of unoccupied patches [14], increase patch turnover [6] and alter the growth rate of other occupied patches depending on density-dependent regulation [9,15]. However, since dispersal causes interdependence in patch dynamics within SSPs [8], any change in the local growth rates or extinction risks of perturbed patches through dispersal, mortality or reduced fecundity [10,12,16,17] may result in additional indirect effects on other patches. For instance, reduced competition following high mortality can increase dispersal toward perturbed patches [18] and decrease biomass in unperturbed patches [19,20]. Depending on the ability of unperturbed patches to absorb such direct and indirect effects, dispersal can act either as a stabilizing or destabilizing process at the SSP level. However, few studies have monitored perturbation effects across multiple patches [14,21,22], and we still know little about how seemingly unaffected patches may be indirectly affected by perturbations and the consequences for overall SSP dynamics.

Consequences of local perturbations at the SSP level can be expected to depend on the number of individuals in the perturbed patch. Patches occupied by large numbers of individuals have a greater ability to withstand stochastic events than patches occupied by smaller numbers [23,24], but perturbing many individuals may also amplify perturbation effects at the SSP level. Even when individuals relocate within the SSP, increased emigration to other patches is likely to be absorbed only up to a point, beyond which saturation and competition occur. Moreover, the more individuals who are removed from the SSP by dispersal and mortality, the greater the reduction in local competition, in the production of dispersers or in social processes shaping dispersal decisions, potentially altering dispersal flows among patches in the longer term. Thus, understanding perturbation effects and the ability of the SSP to recover (i.e. its resilience [25]) requires considering which patches are affected and their importance in structuring the SSP [20,26].

Colonial birds breed in discrete habitat patches and form SSPs exposed to various perturbations, including predator incursions [6,16], human disturbances [11,17] and sudden changes in habitat availability via floods or droughts [16]. Some species can skip reproduction [27], allowing them to buffer punctual perturbations despite high site fidelity. Colonial birds typically disperse when survival or breeding success is threatened [4,12,16]. Moreover, many species rely on social cues, such as conspecific numbers, to guide dispersal [28–31]. Indeed, the benefits of group living, such as reduced predation pressure and increased access to information about foraging and breeding site quality, are enhanced in large colonies [32]. The resulting conspecific attraction shapes dispersal between colonies and is likely to influence both individual responses to perturbations and the resilience of perturbed patches. Although responses of individual colonies to perturbations are well documented, our understanding of the SSP-level consequences and how these are shaped by density dependence in dispersal remains limited [26].

Here, we investigate how the effects of local perturbations on SSP demographics vary with the proportion of the population occupying the perturbed patch in a colonial waterbird. We focus on an SSP of black-headed gulls (*Chroicocephalus ridibundus*) where the periodic draining of ponds occupied by breeding colonies caused recurrent local perturbations. Pond draining generally leads to the extinction of the colony it hosts for 1 year and the redistribution of individuals among other colonies [33]. In this SSP, given the small size of the plain and the large movement ability of the species, dispersal between patches is unlikely to generate any energetic or survival cost, but a negative effect of distance on dispersal has been interpreted as a cost of familiarity loss [34]. Density-dependent philopatry and conspecific attraction further modulate dispersal [31,34]. This SSP showed an unbalanced distribution of individuals, with over half of the breeders historically accumulating on a single pond, La Ronze (LR), which was continuously occupied for over 30 years before being drained for one season in 2007 [33,35]. Before its perturbation, the large LR colony contributed considerably to the production of juveniles settling in smaller colonies and attracted a significant part of dispersing individuals through positive density dependence [35].

We thus expected the SSP to respond differently to the perturbation of LR compared with smaller colonies. Given the frequency of pond draining, we expected the SSP to be resistant to the perturbation of colonies hosting a small fraction of the population, with limited consequences for the distribution of breeding numbers and SSP growth rates, aside from the local relocation of breeders. However, owing to the size of the LR colony and its weight in dispersal dynamics, we expected its perturbation to result in a regional redistribution of breeding numbers, with extended consequences for dispersal shaped by density dependence. We expected limited changes in the SSP growth rate if breeders relocated to other favourable ponds within the SSP.

We first characterized the consequences of pond draining on SSP structure and dynamics across spatial scales, from patch recolonization to population growth rates, to determine whether these consequences depend on the size of the perturbed colony. We then focused on the perturbation that affected the main colony, on LR, and used an integrated population model to quantify its demographic consequences within the SSP. We paid particular attention to dispersal among colonies and its implications for SSP resilience.

## 2. Material and methods

### (a) Study species and system

The black-headed gull is a medium-sized, long-lived and partially migratory colonial bird species occupying a range of wetland habitats, including lakes, rivers and coastal areas across Eurasia. From April to June, individuals breed in colonies ranging from a few to several thousand pairs in freshwater habitats with abundant vegetation. Black-headed gulls have a maximum lifespan exceeding 30 years (mean≃7 years in the study population [35]) and start breeding at between 2 and 5 years of age [36]. Clutch size ranges from one to three eggs (mean≃2.7 in the study population), with biparental incubation and chick rearing [37].

The black-headed gull population breeding on the Forez plain, an agricultural lowland of 20 × 40 km² in central France, has been monitored since 1976 [38]. Each year, colonies settle on vegetation belts and islets of about 10 to 30 ponds (mean ± s.d. = 17 ± 7) within a network of over 350 human-managed ponds (supplementary material 1, figure S1.1). Colonies show moderate to high interannual turnover, resulting in the occupation of 100 different ponds since 1976. This population thus constitutes a clear example of SSP. Occupied ponds are generally about 1 m deep and range from 5500 to 242 000 m² (mean ± s.d. = 73 500 ± 51 500 m²). Although ponds are regularly drained in winter, historically to harvest fish, some occasionally remain empty for 1 year as part of their management. Such draining events extending into gulls’ breeding season can force colony extinction for 1 year if the pond was occupied the previous year [33]. A large pond managed as a nature reserve, LR (45.5751° N/4.2262° E), hosted the largest colony in the SSP for most of the study period. LR colony went extinct in only 1 year, when LR was drained in 2007. The Forez population is known to be connected with other European populations of black-headed gulls, as immigrants have also occasionally been observed [38,39].

### (b) Data collection

Since 1976, annual surveys (terrestrial from 1976 to 1978 and aerial from 1979 onwards) have been conducted to locate all colonies on the plain and identify drained ponds. Ponds were considered drained when at least three-quarters of their bottom surface area was exposed, preventing colony establishment. The number of breeding pairs in each colony was then estimated during terrestrial visits, based on the number of flying adults counted after a brief general take-off was induced (supplementary material 1) [38]. However, on LR pond, the large colony size led to imprecise counts before 2007 (mean = 3230 pairs; range: 2000–5000) compared with other colonies (mean = 165 pairs; range: 1–1600). To address this, we used corrected numbers for LR provided by Grosbois [40]. These ‘corrected counts’ were based on the ratio of ringed to unringed birds, accounting for imperfect ring detection (supplementary material 1). Data required for this correction were only available between 1981 and 2000. Between 2001 and 2006, terrestrial counts for LR still suffered from the same imprecision but indicated stable breeding numbers in this colony. We therefore assumed LR to have a constant size equal to the corrected count of 2000 over this period [35]. From 2007 onward, LR colony size was sufficiently small for terrestrial counts to be considered as reliable as for other colonies. Information on colony size was missing for 4 out of 838 colony-years (<0.5%) owing to difficult access to ponds, but aerial surveys confirmed these colonies hosted no more than a few 10 individuals in a population of thousands. Information on drained ponds was missing for 10 out of the 50 study years (1977, 1984, 1986–1991, 2005 and 2016; supplementary material 1).

Capture–recapture (CR) data were collected annually between 1976 and 2019, initially on the LR colony and, from 1994 onwards, on additional colonies. Following Péron *et al.* [35], analyses were restricted to data posterior to 1986 to balance CR data availability between LR and other colonies. Keeping CR data for LR between 1986 and 1994 reinforced our ability to characterize population dynamics in the early years of the monitoring. Overall, CR data used here covered nine colonies from 1986 to 2019, including LR colony and eight other colonies referred to as ‘satellite colonies’ (supplementary material 1): Les Marquants (MA, 45.7897° N/4.0940° E), La Verchère (VE, 45.8152° N/4.0668° E), La Vallon 5 (V5, 45.6518° N/4.0901° E), La Vergnat Sud (VS, 45.6307° N/4.2000° E), Les Champs Blancs (LA, 45.6190° N/4.1879° E), Sury (SV, 45.6988° N/4.2831° E), Vieux–Ouest (WV, 45.7152° N/4.2507° E) and La Sauzée (SZ, 45.5752° N/4.1668° E). Only the LR colony was monitored by CR every year, whereas recapture effort on satellite colonies varied with colony presence and authorizations to access ponds. Nestlings were captured by hand and ringed with alphanumeric metal rings [38], with additional colour or darvic rings in the last 30 years to ease individual identification from a distance. We also included some nestlings ringed on other colonies of the SSP with occasional ringing effort. In some years, adults were captured and ringed in surrounding ploughed fields and landfills using a cannon net. For the nine colonies with CR data, resighting of breeding individuals were conducted by observers entering the colonies to read rings using telescopes mounted inside floating hides. Between 1986 and 2019, CR data consisted of 33 285 ringed individuals and 6 030 resighting of adult birds on breeding colonies (assumed to be breeders).

### (c) Effect of pond draining on patch and spatially structured population dynamics

We examined the general effect of pond draining on SSP dynamics and its dependence on the size of perturbed colonies using linear models and descriptive statistics. First, at the patch level, we compared recolonization probability after a pond was drained for one season with (i) the persistence probability of unperturbed colonies and (ii) recolonization probability following colony extinction unrelated to draining. Therefore, we here treated two colonies as distinct if they either occupied different ponds, or occupied the same pond but were separated by more than 1 year of extinction. This resulted in 194 distinct colonies over the study period, persisting for an average of 3 years (range: 1–36 years), of which 89 were present in at least 2 years. For colonies present in year t, we fitted a binomial generalized mixed model relating colony presence in year (t+2) to pond state in year (t+1) (drained, not drained but unoccupied, not drained and occupied) and the logarithm of colony size in year t. We considered colony size in year t and not (t+1) to investigate the effect of colony size on occupancy probability in year (t+2) even after 1 year of extinction (i.e. when the colony is absent in year (t+1)). We included colony as a random effect to account for non-independence in the dynamics of colonies persisting for more than 1 year. An interaction between colony size and pond state was initially included but removed as it was not significant. Years lacking data on drained ponds were excluded, assuming they had similar dynamics to the majority of the study period for which we had data on drained ponds.

Second, at the SSP level, we assessed the effect of pond draining on changes in total breeding numbers. We used a linear model relating SSP growth rate between year t and (t+1) to the proportion of SSP breeders occupying ponds in year t that were drained in year (t+1). To test for changes following the draining of LR in 2007, we included an interaction between this proportion and a period factor (before/after 2007). Years with missing data on drained ponds were excluded. All linear models were fitted using R v4.3.2 [41] and the *glmmTMB* package v1.1.11 [42], with marginal means computed using the *emmeans* package v1.11.1 [43].

Third, we described changes in SSP structure by calculating Gini coefficients for colony size using the *ineq* package v0.2-13 [44]. Gini coefficients quantify colony size heterogeneity in each year, with values close to 0 indicating near-equal colony sizes, whereas values close to 1 indicate strong concentration of breeders in a single colony.

### (d) Integrated population model

We characterized changes in survival, fecundity and dispersal following the draining of LR in 2007 using an integrated population model (IPM) similar to that of Péron *et al.* [35], adapted in a Bayesian framework based on model structures from Schaub *et al*. [45]. The IPM was fitted to data from 1986–2019 and combined (i) a population count model and (ii) a multi-state CR model, with states corresponding to age classes within each colony with CR data. As all colonies were assumed to be detected and counted in the SSP, an additional (‘alive elsewhere’, AE) state allowed us to distinguish between dispersal to colonies of the SSP without CR data and long-distance emigration out of the SSP, which cannot be distinguished from mortality.

#### (i) Life-cycle formalization

The life-cycle of black-headed gulls underlying the IPM comprised three stages (supplementary material 2, figure S2.1): nestlings (N), pre-breeders (P) and breeders (B). Pre-breeders and breeders share the same apparent survival probability $\phi_{ad,t}$, distinct from juvenile apparent survival between fledging and first year $\phi_{juv,t}$. Both survival parameters vary annually through a random year effect modelled on the *logit* scale. Only breeders produce nestlings with fecundity $\rho_{i}$, which differs between LR and satellite colonies following previous results [35]. Pre-breeders recruit with probability κ, set to 0 for first-year individuals and to one for individuals aged two or more to address identifiability issues, despite recruitment occurring between ages two and five [35,46]. We assume reproduction skipping to be negligible, so individuals remain breeder once recruited. Dispersal is summarized as average flows of individuals between colonies over periods showing temporally consistent dynamics (see model application). This allowed us to pool information on dispersal over several years and compensate for missing CR data on some colonies in some years, while still enabling the comparison of average dispersal dynamics between different periods. Natal dispersal $\eta_{j,\;i,t}$ from colony j to i in year t is only detectable when recruitment happens, and thus integrates all movements between birth and first reproduction. Breeders can disperse each year t from colony j to i with probability $\nu_{j,\;i,\;t}$. In years where colony i disappears, dispersal towards that colony $\eta_{j,\;i,t}$ and $\nu_{j,\;i,\;t}$ are constrained to zero. The remaining dispersal probabilities from colony j are rescaled to sum to one, assuming no change in relative dispersal among colonies within each period considered to show relatively homogeneous dynamics.

We modelled female abundance in stages P and B in colony i in year (t+1) considering a pre-breeding census. Pre-breeders consist of first-year individuals produced by breeders in the previous year, and surviving pre-breeders that did not recruit:

(2.1) $$P_{i,\;t+1}\;=\;B_{i,t}\;\cdot \rho_{i}\cdot \phi_{juv,t}\;+\;P_{i,t}\cdot \phi_{ad,t}\cdot \left(1-\kappa \right).$$

Breeders consist of surviving pre-breeders that recruited and surviving breeders, considering both natal and breeding dispersal among colonies:

(2.2) $$B_{i,\;t+1}\;=\;\underset{j}{\sum }P_{j,\;t}\cdot \phi_{ad,t}\cdot \kappa \cdot \eta_{j,\;i,t}\;+\;\underset{j}{\sum }B_{j,\;t}\cdot \phi_{ad,t}\cdot \nu_{j,\;i,t}.$$

#### (ii) Population count model

We used a state-space formulation for the population count model. The state process follows the matrix population model described by equations 2.1 and 2.2. The observation process links $B_{i,\;t}$ with colony counts $C_{i,\;t}$ assuming a normal observation error $\sigma_{i,t}$ proportional to colony size, accounting for differences in count precision between small and large colonies. The error is based on a proportionality factor $\sigma_{LR,\;SAT}$ modelled separately for LR and satellite colonies, which notably accounts for differences in precision between terrestrial counts and corrected counts for LR:

(2.3)Ci,t∼Normal(Bi,t,σi,t)(2.4)andσi,t=Bi,t⋅σLR,SAT.

#### (iii) Multi-state capture–recapture model

We defined a total of 31 capture states s to encode individual capture histories: each of the three life stages could be alive in any of the nine colonies with CR data, or alive but undetectable in colonies without CR data (AE). Quantifying dispersal from and toward this AE state accounted for all dispersal events within the population range. The final state represents death or permanent emigration from the SSP.

A transition probability matrix $\Psi_{t}$ describes the probability of moving from state $s_{t}$ in year t to $s_{t+1}$ in year (t+1), based on the species’ life cycle (supplementary material 2, figure S2.1). For example, with three life stages and two colonies i and j, $\Psi_{t}$ has six states:

$$\begin{array}{r} \Psi_{t}=\begin{array}{ccc} & N_{i,t} & \\ & N_{j,t} & \\ & B_{i,t} & \\ & B_{j,t} & \\ & P_{i,t} & \\ & P_{j,t} & \end{array}\begin{array}{cl} & \begin{array}{cllllll} & \;\;\;N_{i,t+1} & \;N_{j,t+1} & \;\;B_{i,t+1} & \;\;\;B_{j,t+1} & \;\;P_{i,t+1} & \;P_{j,t+1}\; \end{array}\\ & \left(\;\begin{array}{cccccccc} \; & 0 & \;0 & \;0 & 0 & \phi_{juv,t} & 0 & \\ \; & 0 & \;0 & \;0 & 0 & 0 & \phi_{juv,t} & \\ \; & 0 & \;0 & \;\phi_{ad,t}.\nu_{i,\;i,t} & \;\phi_{ad,t}.\nu_{i,\;j,t} & 0 & 0 & \\ \; & 0 & \;0 & \;\phi_{ad,t}.\nu_{j,\;i,t} & \;\phi_{ad,t}.\nu_{j,\;j,t} & 0 & 0 & \\ \; & 0 & \;0 & \;\phi_{ad,t}.\kappa .\eta_{i,\;i,t} & \;\phi_{ad,t}.\kappa .\eta_{i,\;j,t} & \;\phi_{ad,t}.\left(1-\kappa \right) & 0 & \\ \; & 0 & \;0 & \;\phi_{ad,t}.\kappa .\eta_{j,\;i,t} & \;\phi_{ad,t}.\kappa .\eta_{j,\;j,t} & 0 & \;\phi_{ad,t}.\left(1-\kappa \right) & \end{array}\;\right) \end{array}\; \end{array}$$

A detection probability matrix $P_{t}$ models our ability to observe individuals in the different states. As only breeding individuals are resighted, nestlings and pre-breeders have detection probability of 0. For example, with three life stages and two colonies i and j, $P_{t}$ has six states:

$$\begin{array}{r} P_{t}\;=\begin{array}{cl} & \begin{array}{llllll} \;N_{i,t} & \;N_{j,t} & \;B_{i,t} & \;B_{j,t} & \;P_{i,t} & \;P_{j,t} \end{array}\\ & \left(\;\begin{array}{llllll} 0 & \;0 & \;p_{i,t} & \;p_{j,t} & \;0 & \;0\; \end{array}\;\right) \end{array}. \end{array}$$

In years t where no recapture effort occurred on colony i, $p_{i,t}$ is set to 0, ensuring that no spurious information is inferred in the absence CR data. Detection probabilities for AE states over the T years of the study period $p_{AE,\;1\;\;:\;T}$ are 0, as no recaptures happened on colonies without CR data by definition. Other $p_{i,t}$ include a random year effect on the *logit* scale to account for interannual variation in observation effort.

Individual capture histories were summarized in a two-dimensional m-array $M_{(T-1)\times S,\;(T-1)\times S}$, where each cell counts individuals last captured in state $s_{t_{c}}$ in year $t_{c}\in \;[1\;:(T-1)]$ and recaptured in state $s_{t_{r}}$ in year $t_{r}\in \;[\;(t_{c}+1)\;:T]$. Each row was modelled with a multinomial distribution, involving $\Psi_{t}$, $P_{t}$ and the number of individuals released in state $s_{c}$ in year $t_{c}$ [45].

### (e) Model implementation

The IPM was implemented in a Bayesian framework using R v4.3.2 [41] and JAGS v4.3.1 [47] via the *jagsUI* package v1.6.2 [48]. CR data were formatted as m-arrays using the *IPMbook* package v0.1.5 [49]. We used non-informative priors for all parameters (supplementary material 2). Model convergence was assessed through visual inspection of the MCMC chains and a Gelman–Rubin convergence statistic below 1.1 [50]. Model performance was validated with simulated data (supplementary material 2).

### (f) Model application and post hoc analyses

To assess the impact of LR draining, we run the IPM on four time periods defined from a preliminary exploration of the data: the period before the perturbation (1986–2006), as the initial state with a relatively stable population structure and growth rate [35]; the period around the perturbation (2006–2007), to characterize the immediate effects of the extinction of LR colony; the period directly following the perturbation (2007–2010), as a potential short-term transition leading to SSP recovery; and a medium-term post perturbation period (2011–2019), when SSP dynamics were expected to have stabilized. Over each period, population dynamics appeared sufficiently homogeneous for the assumption of constant relative dispersal to be reasonable. This assumption was relaxed between periods as the IPM was run separately over each, enabling the estimation of independent demographic parameters, including dispersal. With only two years of data for the period around the perturbation (2006–2007), the IPM could not estimate most parameters for this particular period, but was still run to estimate annual survival in the year of the perturbation from count data. The definition of the short-term post-perturbation period (2007–2010) reflected a trade-off between data sufficiency for precise parameter estimation and the risk of diluting a potentially brief demographic signal over a too long interval.

Not all colonies were subject to CR during all periods. Capture states included four colonies from 1986–2006 (LR, MA, VE and V5), six colonies from 2006–2010 (LR, MA, VS, LA, SV and WV), and seven colonies from 2011–2019 (LR, MA, VS, LA, SV, WV and SZ).

Post hoc analyses were carried out on IPM outputs to investigate the role of density dependence in shaping dispersal before and after LR draining. Spearman correlations were computed between posterior dispersal estimates and average colony size estimates for each period using the *stats* package v4.4.3 [41], using the entire posterior distributions of parameters to fully propagate uncertainty. Correlations between natal or breeding fidelity and colony size indicate density-dependent emigration, while positive correlations between natal or breeding settlement and colony size indicate conspecific attraction.

## 3. Results

### (a) Characterization of the perturbation regime

On average, 1.0 occupied ponds were drained each year over the study period (range: 0–3), producing multiple perturbations of varying magnitude (figure 1). Before 2007, colonies occupying a pond that was drained in the following year hosted on average 2.6% of breeders (s.d. = 2.6%, range: 0.1–9.1%). The draining of LR thus generated a perturbation of exceptional magnitude, as this pond hosted 64% of the breeders of the SSP in the preceding year. After 2007, perturbed colonies hosted an average of 10.4% of breeders with greater variability (s.d. = 12.5%, range: 0.1–44.4%), including 1 year when over 50% of breeders were affected by simultaneous draining of two ponds occupied the year before, in 2015.

**Figure 1.**
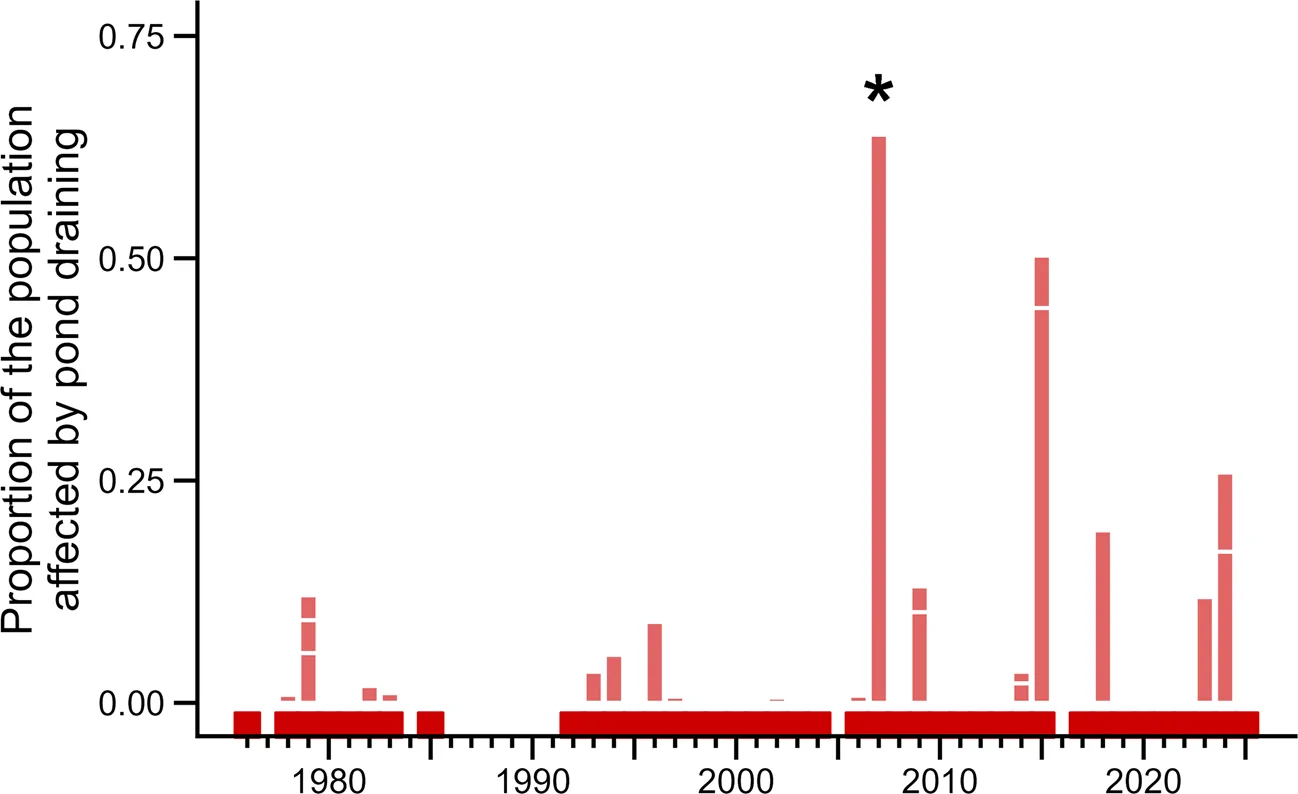
Proportion of the breeding population occupying ponds the year before they were drained. Each stacked bar corresponds to an event in which a pond occupied by a colony in the previous year was drained. Values on the *y*-axis correspond to the proportion of total breeders that occupied the pond in the year before it was drained. The draining of LR in 2007 is marked by a black asterisk. Red rectangles on the x-axis show years with data on drained ponds.

### (b) Consequences of pond draining on patch dynamics

Pond occupancy depended on colony presence in the previous year (supplementary material 3, figure S3.1). The marginal mean for recolonization probability after 1 year of extinction owing to pond draining was 0.52 [95% confidence interval: 0.32; 0.71], not statistically different from recolonization after extinction from other causes (unoccupied pond: 0.36 [0.26; 0.47], *Z* = 1.435, *p* = 0.45). However, it was significantly lower than the persistence probability when a colony was present in the previous year (occupied pond: 0.84 [0.79; 0.87], *Z* = –3.372, *p* < 0.01). Both persistence and recolonization probabilities increased with the size of the colony that occupied the pond two years before, i.e. before the potential extinction of the colony (mean coefficient ± SE = 0.43 ± 0.08, *Z* = 5.470, *p* < 0.001).

### (c) Consequences of pond draining on spatially structured population dynamics and structure

Colony counts and IPM estimates revealed three phases in the population dynamics (figure 2). Between 1976 and the mid-1980s, breeding numbers rose from 5000 breeding pairs to about 9000 pairs, coinciding with an increase in the size and number of satellite colonies visible from the difference between LR counts and total population size (figure 2). Between 1989 and 2006, count data and IPM estimates showed a median decline of 36% [95% credible interval: 2%; 53%]. After 2007, numbers declined at a similar rate, falling 35% [12%; 51%] over eleven years. No relationship was found between SSP growth rates and the proportion of the population affected by pond draining before 2007 (mean coefficient ± SE = –1.49 ± 1.71, *Z* = –0.867, *p* = 0.39), nor after 2007 (mean difference between periods ± SE = 0.79 ± 1.77, *Z* = 0.444, *p* = 0.66), suggesting no effect of the draining of ponds occupied by small or medium-sized colonies on population dynamics.

**Figure 2.**
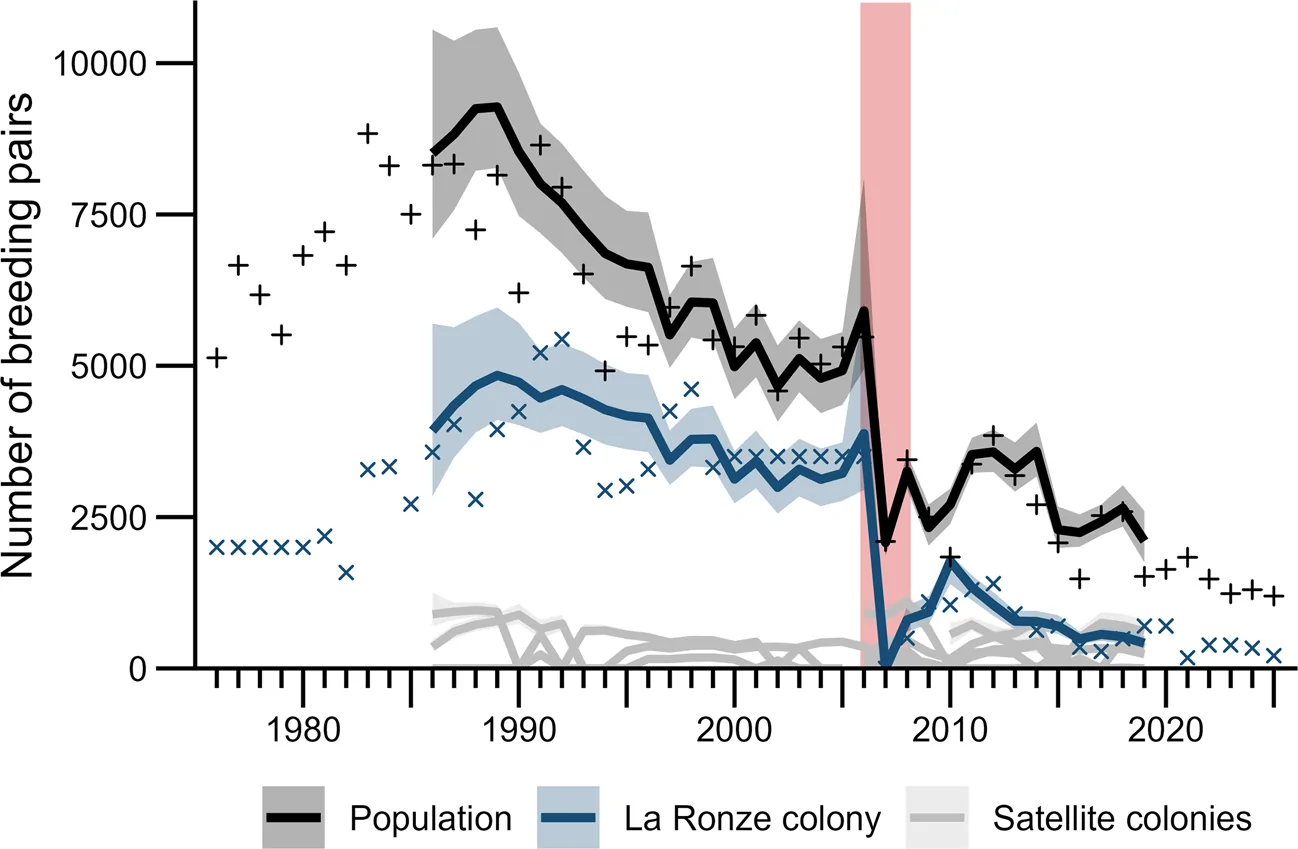
Number of breeding pairs in the colonies monitored by CR from 1976 to 2025. Crosses correspond to raw count data from terrestrial surveys and ‘corrected counts’ re-estimated from ringing data for the LR colony for 1981–2006. Lines and shaded areas represent the estimated means and 95% credible intervals from the integrated population models for the number of breeders in the SSP (population, black), in LR colony (blue) and in each satellite colony (grey, one line for each colony with CR data). The red vertical band highlights the draining of LR in 2007.

However, the draining of LR in 2007, which hosted an estimated 3900 [2947; 6080] pairs in 2006, coincided with a sharp decline of 65% [54%; 77%] in total breeding numbers. After this event, the LR colony recovered only partially, with an estimated 800 [606; 1035] pairs in 2008, and annual numbers fluctuating between 400 and 1800 since then. Total breeding numbers fell from an estimated 5900 [4943; 8096] pairs in 2006 to about 3250 [2972; 3542] pairs in 2008, and have remained between 2100 and 3600 since then.

Regarding SSP structure, the annual Gini coefficient of colony sizes decreased from an average of 0.69 (s.d. = 0.08) before 2007 to 0.54 (s.d. = 0.11) after 2007, indicating homogenization of colony sizes following the draining of LR (supplementary material 3, figure S3.2).

### (d) Variation in survival and fecundity induced by the draining of La Ronze

The draining of LR coincided with a change in SSP demographic rates (figure 3). Mean adult apparent survival dropped from 0.84 [0.79; 0.88] in 1986–2006 to 0.26 [0.20; 0.34] in 2006–2007 (supplementary material 3, figure S3.3). Subsequently, it increased to 0.76 [0.56; 0.90] in 2007–2010 before returning to pre-perturbation levels in 2010–2019 (median: 0.87 [0.78; 0.93]). However, interannual variability increased after the perturbation, with adult survival notably dropping below 0.60 in 2010 and 2015 (figure 3).

**Figure 3.**
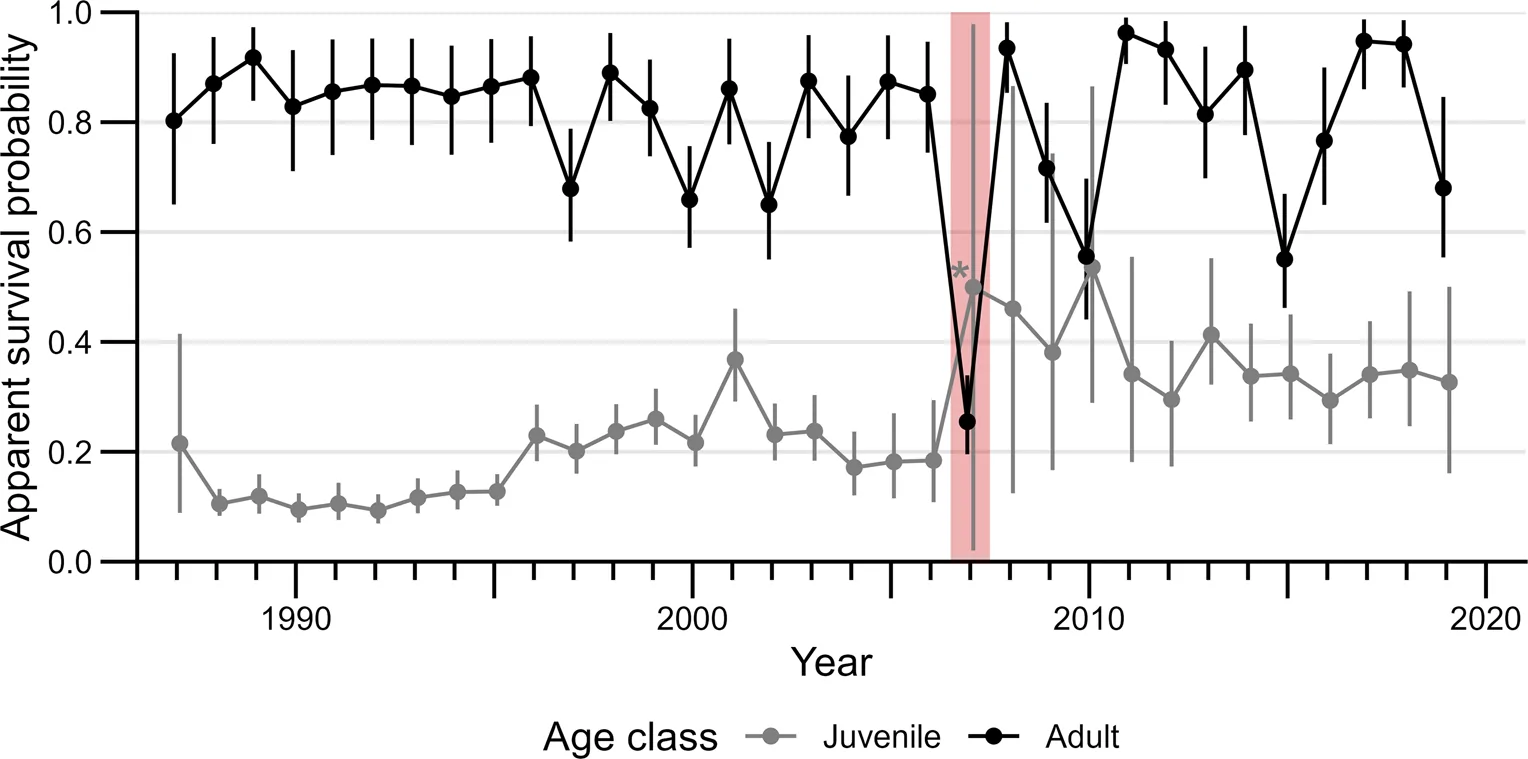
Interannual variation in apparent survival of the black-headed gulls over the study period. Dots and error bars show median and 95% credible intervals of posterior distributions. Values for adults and pre-breeders are in dark and values for juveniles are in grey. The vertical band marks the perturbation that occurred at LR in 2007. The grey asterisk marks the estimate of juvenile survival in 2007, which was non-identifiable by the model.

Juvenile survival in the year of the perturbation could not be estimated (supplementary material 3, figure S3.3). Estimates were also imprecise in 2007–2010. However, mean juvenile apparent survival increased from 0.17 [0.14; 0.22] in 1986–2006 to 0.34 [0.26; 0.42] in 2010–2019, with a gradual increase already visible since the 1990s (figure 3).

As an uninformed parameter in the IPM, fecundity was not properly estimated. Between 1986 and 2006, estimates hit the upper limit of their prior distribution at 0.97 [0.91; 1.00] for both LR and satellite colonies (supplementary material 3, figure S3.4). Large credibility intervals after 2006 suggest that fecundity absorbed much of the unmodelled variance.

### (e) Variation in dispersal parameters induced by the draining of La Ronze

Patch fidelity and settlement probabilities changed after the draining of LR (figure 4). Breeding fidelity to LR decreased from 0.98 [0.97; 0.99] in 1986–2006 to 0.58 [0.50; 0.65] in 2010–2019, while natal fidelity to LR decreased from 0.78 [0.73; 0.83] to 0.25 [0.15; 0.37]. Fidelity to satellite colonies also decreased following the perturbation, although these differences should be interpreted cautiously as satellite colonies differ between periods, except MA, which was occupied during all three periods (figure 4a,b).

**Figure 4.**
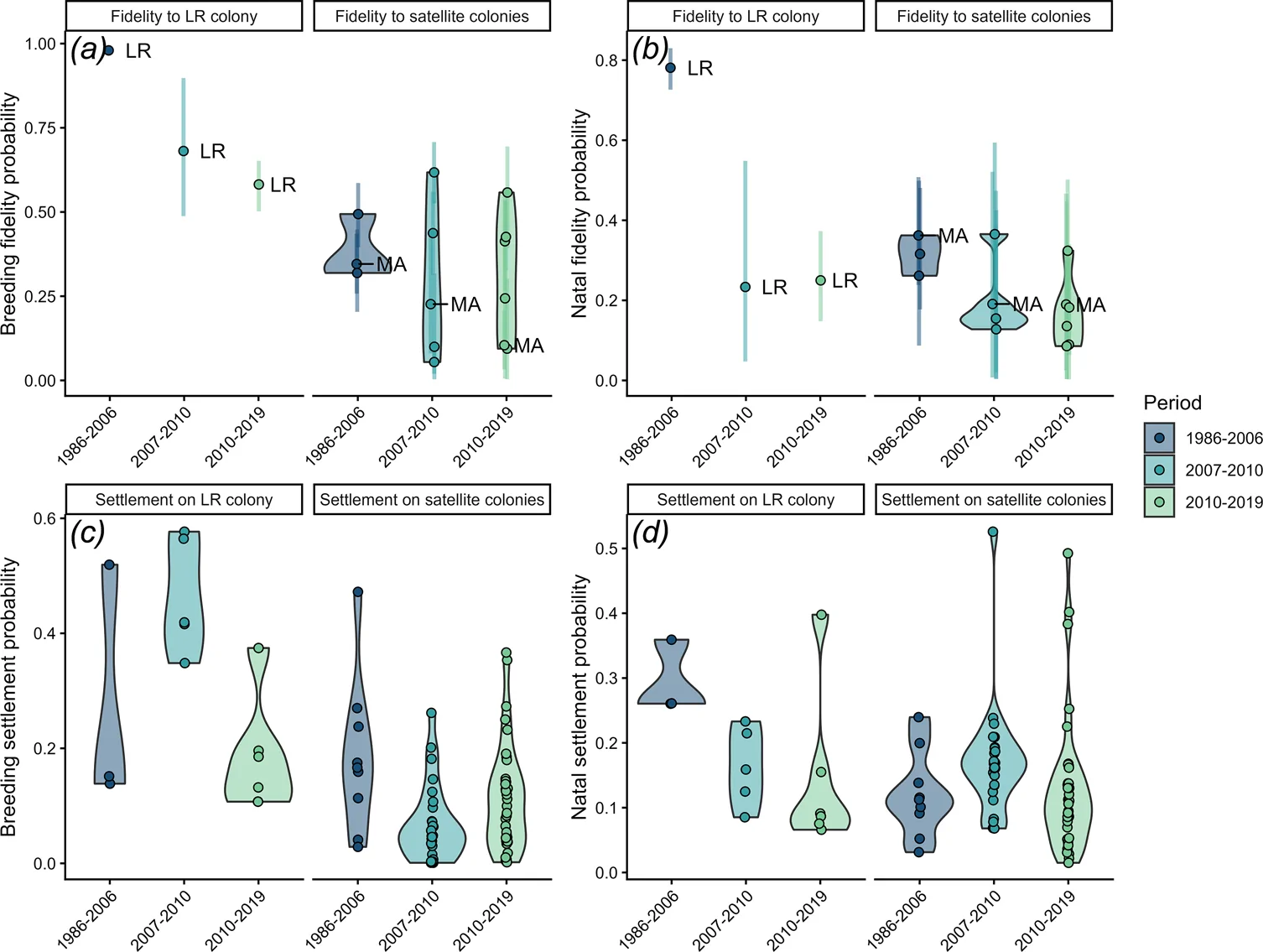
Variation in dispersal parameters between colonies over the study period. (a) Mean fidelity for breeding dispersal and (b) natal dispersal, for LR colony (left) and satellite colonies (right), including MA. (c) Mean settlement probability for breeding dispersal and (d) natal dispersal, conditional on individuals not remaining in the same colony, for LR (left) and satellite colonies (right). (a,b) 95% credibility intervals are only displayed for fidelity to avoid overcharging the plots. Data points for each combination of departure and destination colony are displayed with a small jitter within the violin plots. Results are shown for three of the study periods: before the perturbation (1986–2006, dark blue), just after the perturbation (2007–2010, light blue) and in the mid-term after the perturbation (2010–2019, green).

Mean breeding settlement probability on LR increased from 0.14–0.52 to 0.34–0.58 in 2007–2010, but returned to pre-perturbation levels in 2010–2019 (figure 4c). By contrast, mean natal settlement on LR decreased overall from 0.26–0.36 to 0.07–0.40 after the perturbation, with a few satellite colonies attracting more natal dispersers than LR from 2007 onwards (figure 4d).

### (f) Correlation between dispersal parameters and colony size

Spearman correlations between dispersal and colony size were positive in most periods (supplementary material 3, figure S3.5). Breeding fidelity correlated similarly with colony size before and after the perturbation (0.66 [0.40; 0.80] in 1986–2006 and 0.76 [0.43; 0.96] in 2010–2019), peaking just after the perturbation in 2007–2010 (0.92 [0.83; 1.00]). Natal fidelity was positively correlated with colony size before the perturbation (0.79 [0.20; 1.00] in 1986–2006), but not after (0.20 [–0.60; 0.83] in 2007–2010 and 0.52 [–0.11; 0.93] in 2010–2019), although differences between periods were not significant.

Breeding settlement probabilities were consistently positively correlated with colony size across all periods (0.51 [0.24; 0.80] in 1986–2006, 0.65 [0.49; 0.79] in 2007–2010 and 0.47 [0.31; 0.63] in 2010–2019). Natal settlement was positively correlated with colony size before and after the perturbation (0.72 [0.43; 0.91] in 1986–2006 and 0.31 [0.09; 0.51] in 2010–2019), but not immediately after 2007 (0.05 [–0.29; 0.39] in 2007–2010), although differences between periods were not significant.

## 4. Discussion

In our study system, the temporary draining of ponds regularly induced perturbations affecting a small proportion of breeders (<10% before 2007). Such events caused the extinction of the affected colonies and reduced the occupancy probability of these perturbed patches in the following year, but had no detectable effect on population size or SSP demographic parameters. However, the perturbation of the main colony of LR in 2007, which hosted 64% of the breeders at that time, had a strong and lasting impact on SSP structure and dynamics. Breeding numbers fell sharply in the year of the perturbation and never recovered, and colony sizes became more homogeneous. Apparent adult survival decreased at the time of the perturbation and has since shown increased interannual variability. Dispersal dynamics also became more uniform after the perturbation, although the positive correlation between colony size and dispersal rates persisted.

### (a) General response of the spatially structured population to pond draining

Three non-exclusive responses to pond draining could have been expected: temporary reproduction skipping with site fidelity, relocation of breeders within the SSP, or long-distance emigration from the SSP. In our system, pond drainings were frequent but generally caused low-magnitude perturbations, affecting on average 2.6% of the breeders before 2007 and 10.4% after. Consistent with previously described ‘displacement-fragmentation’ dynamics of colonies that disappear [33], the main response to pond draining seemed to be relocation to unperturbed patches—likely nearby ones, given dispersal in this population is distance dependent [34]. This had limited consequences for population dynamics. Indeed, SSP growth rates were not correlated with the proportion of breeders affected, indicating that most individuals remained within the SSP when small or medium-sized colonies were perturbed. Pond draining affects only the availability of a handful of breeding sites each season, and the Forez plain numbers at least 350 medium-to-large ponds that could host colonies, including 100 ponds occupied at least once since the beginning of the monitoring. Relocation to unperturbed patches appears to be the typical response of colonial birds to localized perturbations [4,12,16,51] and black-headed gulls are no exception.

At the patch level, pond draining caused colony extinction and reduced occupancy probability the following year compared with patches occupied by persistent, unperturbed colonies. Patch resilience to pond draining thus appeared to be limited, contributing to patch occupancy turnover. Perturbations caused by repeated predator incursions resulted in comparable patch dynamics in Audouin's gulls (*Ichthyaetus audouinii*) [6]. However, recolonization rates were comparable to those after other extinction causes, which, if not reflecting low statistical power, could suggest that pond draining did not elicit exceptional colony responses. The overall functioning of the SSP remained unchanged, suggesting little sensitivity of the population to low-magnitude, frequent pond draining, which can be considered part of the system's inherent dynamics. Whether the population exhibits resistance to low-magnitude perturbations and quickly recovers to perturbation-independent dynamics, or is maintained in continuous transitional recovery by the high frequency of perturbations, remains to be determined. This ambiguity has implications for defining equilibrium dynamics in populations subject to repeated perturbations and for our understanding of population functioning, given that demographic drivers operating during recovery are not necessarily those governing non-recovery dynamics.

### (b) Immediate response to the perturbation of La Ronze: permanent emigration from the spatially structured population

The draining of LR forced the extinction of the main colony that hosted over 60% of breeders. Unlike the perturbation of smaller colonies, a perturbation of this magnitude seems to have crossed a threshold and had direct consequences at the SSP level. Breeding numbers dropped by 65% in the year of the perturbation, with no subsequent recovery, coinciding with a more than threefold reduction in adult apparent survival. Since black-headed gulls are long-lived and the perturbation only affected the availability of one breeding site, this apparent survival drop likely reflects mass long-distance emigration [52]. We know this emigration was directed out of the SSP and not towards colonies of the SSP without CR data, as we modelled an ‘alive elsewhere’ state [35]. Most emigrants were likely breeders from LR, as the perturbation was localized and LR concentrated most of the breeding numbers. However, since we ran the model separately for each period to accommodate significant changes in dynamics following the perturbation, we were unable to account for individuals that may have returned to the SSP several years later. Yet, this exceptional emigration response suggests that the impact of a perturbation on SSP dynamics depends strongly on the number of individuals in the perturbed patch. The large disparity in colony size between LR and the other perturbed colonies prevented identification of the proportion of total population above which local perturbations propagate to the population level. Such a threshold, certainly population-dependent, could be estimated through simulation studies or inferred from continued monitoring, as colonies now hosting a larger share of breeders may themselves experience perturbations in coming years. Also, as only one satellite colony (MA) was present and monitored by CR both before and after the perturbation, our ability to understand the consequences of LR perturbation on the size and composition of other colonies remains limited (supplementary material 3, figureS3.6).

Several factors could explain why the draining of LR triggered long-distance emigration rather than relocation within the SSP. Breeding numbers had been declining before this perturbation [35], and we found that this trend has continued since then. Concurrent changes in agricultural practices and landfill closures are suspected to have reduced foraging resources and the SSP carrying capacity [53]. Notably, gulls historically foraged at two landfills located within 10 km of LR [54], which probably contributed to the accumulation of breeders on LR alongside the stability and abundance of nesting sites on that pond, until these landfills closed in the 1990s. Similar observations were made in yellow-legged gulls (*Larus michahellis*), for which landfill closure negatively affected the apparent survival of first-year individuals [55]. In our system, conspecific attraction to the large LR colony may have slowed emigration out of the SSP despite deteriorating local conditions. The draining of LR could have removed this attraction, and small colonies may not have been able to attract or accommodate the high number of breeders attempting to relocate from LR. This hypothesis emphasizes the role of background population dynamics in shaping responses to perturbations and predicting SSP vulnerability. Yet whether the perturbation of LR would have truly had different consequences under stable population dynamics remains an open question.

### (c) Lasting consequences of the perturbation of La Ronze on spatially structured population demographics

Breeding numbers in the SSP declined at similar rates before and after the perturbation of LR, which could suggest no lasting change in overall population dynamics, consistent with previous findings in other long-lived bird species [17]. However, adults and juveniles responded differently to the perturbation, pointing to persistent effects on underlying demographic rates. Juvenile apparent survival increased, likely reflecting reduced juvenile emigration, which may have started even before the perturbation. Juveniles are less competitive for breeding sites, often recruiting in small colonies before gaining access to their natal colony [34]. The increase in juvenile apparent survival may, thus, reflect reduced competition for nesting sites owing to declining breeding numbers in the SSP, compounded by the mass emigration triggered by the perturbation of LR. By contrast, adult apparent survival recovered to pre-perturbation levels, but showed greater interannual variability after the perturbation. Such differences between adults and juveniles could be explained by a greater sensitivity of adults to the quality of breeding sites and to their temporal variability. While differences in the response of young and experienced individuals have led to changes in population age structure in other studies [4,6], our IPM estimates do not suggest a change in the proportion of pre-breeders after the perturbation (supplementary material 3, figure S3.7).

In the absence of productivity data, our IPM was unable to estimate fecundity, which, as an uninformed parameter, absorbed much of the residual variance in the model, including that owing to unobserved immigration [56]. However, the increase in juvenile apparent survival combined with the stability of SSP growth rates and pre-breeder proportions suggests that fecundity likely decreased post-perturbation. Even when individuals return, reproductive output can indeed be reduced for an extended period after a perturbation [10,12]. The mass emigration following the perturbation of LR may have further lowered reproductive success, as breeding success in colonial species depends on colony size [57], although more data are needed to verify this hypothesis.

### (d) Consequences of the perturbation of La Ronze on dispersal dynamics in the spatially structured population

We expected a temporary change in dispersal dynamics following the perturbation of LR, owing to the extinction of the main colony of the SSP and the relocation of part of its breeders. The permanent long-distance emigration we observed instead was accompanied by lasting changes in dispersal dynamics. Assuming constant relative dispersal within each period implied lower resolution for detecting changes between periods, as dispersal was averaged over multiple years. Nevertheless, changes in dispersal dynamics between periods were still strong enough to be detected. Despite the small number of colonies with CR data, colony fidelity generally decreased after the perturbation of LR, with both natal and breeding fidelity to LR decreasing the most. Fidelity to LR reached levels comparable to those of satellite colonies after the colony reformed. This persistent increase in dispersal rates after the perturbation mirrors patterns reported in other SSPs following punctual or sustained perturbations [6,12,17]. Settlement probabilities also became more similar between LR and satellite colonies after the perturbation, suggesting a more even redistribution of dispersers than before. Such changes were not detected after the draining of smaller ponds in a previous study [39]. Despite these changes, colony size remained positively correlated with fidelity and settlement probabilities, with comparable strength before and after the perturbation. This confirms that positive density-dependent philopatry and conspecific attraction continue to structure dispersal in the SSP [31,34]. The observed changes therefore likely reflect the reduction and homogenization of colony sizes rather than changes in dispersal mechanisms. Consideration of behavioural mechanisms thus seems essential to understand how perturbation-induced changes in the structure of an SSP determine changes in dispersal dynamics.

### (e) Resilience of a spatially structured population after a major perturbation

In SSPs, resilience can be characterized at the level of the perturbed patches and of the whole SSP, whose structure and dynamics may or may not recover from the perturbation. At both levels, resilience should theoretically be favoured by strong dispersal dynamics, which enable rescue effects from surrounding patches [20]. Yet our study system showed limited resilience to LR perturbation, despite strong dispersal dynamics that were further amplified by the general decline in site fidelity following the perturbation. At the patch level, the LR colony recovered only about a quarter of its previous breeding numbers in the year following the perturbation—a low degree of patch recovery common in colonial species following punctual perturbations [10–12]. At the SSP level, the persistent changes in colony size distribution and dispersal dynamics suggest that the SSP as a whole was not resilient either to the perturbation of its largest colony. This apparent contradiction between resilience theory and our observations likely results from the same two factors that explain the exceptional response to the perturbation of LR. First, the perturbation affected the patch that played a structuring role in the SSP through density-dependent dispersal processes, which can reduce patch recovery [26]. Indeed, the absence of breeders on LR during the perturbation likely reduced both conspecific attraction and the availability of social information on patch quality in the following year [30], deterring potential recolonization despite the high philopatry of the species [34]. Second, the population had been declining for two decades when the perturbation occurred. In an SSP, the multiplicity of patches may provide redundancy that favours rescue effects or enables the formation of new patches with similar features to the perturbed ones, depending on resource distribution and dispersal dynamics. However, compared with a growing or stable SSP, a declining SSP may lack the environmental conditions required for such redundancy—and to maintain site fidelity—thereby precluding the re-establishment of SSP structure and dynamics after a perturbation. We also assumed that pond draining did not durably alter habitat quality, but subtle changes in pond structure and vegetation may have further reduced the attractiveness or carrying capacity of LR.

As the response of SSPs to perturbations depends on their structure and dynamics, SSP restructuring following a first perturbation could feed back on its long-term persistence, including its ability to respond to and recover from subsequent perturbations. Here, the homogenization of colony sizes following the perturbation of LR may have increased SSP sensitivity to future draining, as a larger fraction of breeders now occupies satellite ponds that are all potentially subject to draining. For instance, the marked decline in adult apparent survival in 2015 coincided with the draining of WV, which hosted about 45% of breeding pairs in 2014. This suggests a perturbation of comparable magnitude to that of LR in terms of the proportion of the population affected and the immediate demographic impact. Increased sensitivity to pond draining could also explain the greater variability in apparent survival following the LR perturbation. Beyond recovery in population size, demographic trends or broad structural features, the concept of resilience in SSPs should therefore ideally encompass an assessment of changes in movement dynamics and their consequences for long-term population persistence. In this regard, a demographic decomposition of SSP dynamics is extremely valuable.

Our results show that the effects of a perturbation affecting a single patch on the structure and dynamics of the entire SSP can strongly depend on the proportion of the population affected. Dispersal can enable the SSP to withstand perturbations of patches occupied by few individuals by allowing them to relocate to undisturbed patches. However, it can also amplify the effects of perturbations affecting highly populated patches when individuals leave the SSP. This can subsequently lead to strong and long-lasting changes in SSP structure and demography. The importance of patch characteristics in determining the impact of perturbations on ecological systems, particularly patch connectivity, is well supported by network theory [58]. Here, we provide additional insight into how patch characteristics influence the response of populations to perturbations via dispersal dynamics. These results highlight the need to explicitly consider dispersal mechanisms and their dependence on population context to understand the impact of perturbations on SSPs and their sensitivity to successive perturbations. Such knowledge of the mechanisms underlying SSP dynamics can only be achieved through long-term monitoring programmes across multiple population patches [59], which will prove invaluable in anticipating the impacts of global changes on animal populations.

## Supplementary Material



## Data Availability

Data and code to reproduce the analyses are available on Zenodo [60]. Supplementary material is available online [61].

## References

[1] Oro D . 2020 Perturbation, behavioural feedbacks, and population dynamics in social animals: when to leave and where to go. Oxford, UK: Oxford University Press.

[2] Frederiksen M , Daunt F, Harris MP, Wanless S. 2008 The demographic impact of extreme events: stochastic weather drives survival and population dynamics in a long-lived seabird. J. Anim. Ecol. 77, 1020–1029. (doi:10.1111/j.1365-2656.2008.01422.x) 18557956

[3] Barros Á , Álvarez D, Velando A. 2014 Long-term reproductive impairment in a seabird after the prestige oil spill. Biol. Lett. 10, 20131041. (doi:10.1098/rsbl.2013.1041) PMC4013692 24789139

[4] Oro D , Pradel R, Lebreton J-D. 1999 Food availability and nest predation influence life history traits in Audouin's gull, *Larus audouinii*. Oecologia 118, 438–445. (doi:10.1007/s004420050746) 28307411

[5] Götmark F . 1992The effects of investigator disturbance on nesting birds. In Current ornithology (ed. DM Power), p. 63–104. Boston, MA: Springer US.

[6] Payo-Payo A , Sanz-Aguilar A, Genovart M, Bertolero A, Piccardo J, Camps D, Ruiz-Olmo J, Oro D. 2018 Predator arrival elicits differential dispersal, change in age structure and reproductive performance in a prey population. Sci. Rep. 8, 1971. (doi:10.1038/s41598-018-20333-0) PMC5792507 29386550

[7] Bowne DR , Bowers MA. 2004 Interpatch movements in spatially structured populations: a literature review. Landsc. Ecol. 19, 1–20. (doi:10.1023/B:LAND.0000018357.45262.b9)

[8] Hanski I . 1998 Metapopulation dynamics. Nature 396, 41–49. (doi:10.1038/23876)

[9] Bosch M , Oro D, Cantos FJ, Zabala M. 2000 Short-term effects of culling on the ecology and population dynamics of the yellow-legged gull. J. Appl. Ecol. 37, 369–385. (doi:10.1046/j.1365-2664.2000.00501.x)

[10] Southern LK , Southern WE. 1982 Effect of habitat decimation on ring-billed gull colony- and nest-site tenacity. Auk 99, 328–331. (doi:10.1093/auk/99.2.328)

[11] Cairns DK , Dibblee R, Daoust P-Y. 1998 Displacement of a large double-crested cormorant, *Phalacrocorax auritus*, colony following human disturbance. Can. Field Nat. 112, 520–522. (doi:10.5962/p.358462)

[12] Fairweather JA , Coulson JC. 1995 The influence of forced site change on the dispersal and breeding of the black-legged kittiwake *Rissa tridactyla*. Col. Waterbirds 18, 30–40. (doi:10.2307/1521396)

[13] Spendelow JA , Monticelli D, Nichols JD, Hines JE, Nisbet ICT, Cormons G, Hays H, Hatch JJ, Mostello CS. 2016 Roseate tern breeding dispersal and fidelity: responses to two newly restored colony sites. Ecosphere 7, e01510. (doi:10.1002/ecs2.1510)

[14] Payo-Payo A , Genovart M, Sanz-Aguilar A, Greño JL, García-Tarrasón M, Bertolero A, Piccardo J, Oro D. 2017 Colonisation in social species: the importance of breeding experience for dispersal in overcoming information barriers. Sci. Rep. 7, 42866. (doi:10.1038/srep42866) PMC5314353 28211483

[15] Igual JM , Forero MG, Gomez T, Oro D. 2007 Can an introduced predator trigger an evolutionary trap in a colonial seabird? Biol. Cons. 137, 189–196. (doi:10.1016/j.biocon.2007.02.003)

[16] Burger J . 1982 The role of reproductive success in colony-site selection and abandonment in black skimmers (*Rynchops niger*). Auk 99, 109–115. (doi:10.2307/4086026)

[17] Oro D , Jiménez J, Curcó A. 2012 Some clouds have a silver lining: paradoxes of anthropogenic perturbations from study cases on long-lived social birds. PLoS ONE 7, e42753. (doi:10.1371/journal.pone.0042753) PMC3425592 22936988

[18] Votier SC , Birkhead TR, Oro D, Trinder M, Grantham MJ, Clark JA, McCleery RH, Hatchwell BJ. 2008 Recruitment and survival of immature seabirds in relation to oil spills and climate variability. J. Anim. Ecol. 77, 974–983. (doi:10.1111/j.1365-2656.2008.01421.x) 18624739

[19] Gilarranz LJ , Rayfield B, Liñán-Cembrano G, Bascompte J, Gonzalez A. 2017 Effects of network modularity on the spread of perturbation impact in experimental metapopulations. Science 357, 199–201. (doi:10.1126/science.aal4122) 28706071

[20] Zelnik YR , Arnoldi J-F, Loreau M. 2019 The three regimes of spatial recovery. Ecology 100, e02586. (doi:10.1002/ecy.2586) PMC6375383 30556129

[21] Fernández‐Chacón A *et al.* 2013 When to stay, when to disperse and where to go: survival and dispersal patterns in a spatially structured seabird population. Ecography 36, 1117–1126. (doi:10.1111/j.1600-0587.2013.00246.x)

[22] Smith JNM , Taitt MJ, Rogers CM, Arcese P, Keller LF, Cassidy ALEV, Hochachka WM. 1996 A metapopulation approach to the population biology of the Song Sparrow *Melospiza melodia*. IBIS 138, 120–128. (doi:10.1111/j.1474-919X.1996.tb04318.x)

[23] Dugger KM , Ainley DG, Lyver PO, Barton K, Ballard G. 2010 Survival differences and the effect of environmental instability on breeding dispersal in an Adélie penguin meta-population. Proc. Natl Acad. Sci. U.S.A. 107, 12 375–12 380. (doi:10.1073/pnas.1000623107) PMC2901434 20566874

[24] Lande R . 1993 Risks of population extinction from demographic and environmental stochasticity and random catastrophes. Am. Nat. 142, 911–927. (doi:10.1086/285580) 29519140

[25] Holling CS . 1973 Resilience and stability of ecological systems. Annu. Rev. Ecol. Syst. 4, 1–23. (doi:10.1146/annurev.es.04.110173.000245)

[26] Wilson KL *et al.* 2023 The role of spatial structure in at-risk metapopulation recoveries. Ecol. Appl. 33, e2898. (doi:10.1002/eap.2898) 37303288

[27] Sanz-Aguilar A , Tavecchia G, Genovart M, Igual JM, Oro D, Rouan L, Pradel R. 2011 Studying the reproductive skipping behavior in long-lived birds by adding nest inspection to individual-based data. Ecol. Appl. 21, 555–564. (doi:10.1890/09-2339.1) 21563585

[28] Boulinier T , McCoy KD, Yoccoz NG, Gasparini J, Tveraa T. 2008 Public information affects breeding dispersal in a colonial bird: kittiwakes cue on neighbours. Biol. Lett. 4, 538–540. (doi:10.1098/rsbl.2008.0291) PMC2610090 18647711

[29] Buxton VL , Enos JK, Sperry JH, Ward MP. 2020 A review of conspecific attraction for habitat selection across taxa. Ecol. Evol. 10, 12 690–12 699. (doi:10.1002/ece3.6922) PMC7713925 33304487

[30] Forbes LS , Kaiser GW. 1994 Habitat choice in breeding seabirds: when to cross the information barrier. Oikos 70, 377–384. (doi:10.2307/3545775)

[31] Gregory KA , Francesiaz C, Barnagaud J-Y, Crochet P-A, Lebreton J-D, Besnard A, Papaïx J. 2026 Social cues from neighboring patches influence patch dynamics in a spatially structured population of a colonial bird. Ecology 107, e70349. (doi:10.1002/ecy.70349) PMC13015182 41879260

[32] Danchin E , Wagner RH. 1997 The evolution of coloniality: the emergence of new perspectives. Trends Ecol. Evol. 12, 342–347. (doi:10.1016/S0169-5347(97)01124-5) 21238100

[33] Lebreton J-D . 1984Eléments sur la biologie de la population forézienne de Mouette rieuse. In Paysages et milieux de la plaine du forez, (ed. F Tomas), pp. 293–310. Saint-Etienne, France: Centre d’études foréziennes.

[34] Péron G , Lebreton J-D, Crochet P-A. 2010 Breeding dispersal in black-headed gull: the value of familiarity in a contrasted environment. J. Anim. Ecol. 79, 317–326. (doi:10.1111/j.1365-2656.2009.01635.x) 19891713

[35] Péron G , Crochet P-A, Doherty Jr. PF, Lebreton J-D. 2010 Studying dispersal at the landscape scale: efficient combination of population surveys and capture–recapture data. Ecology 91, 3365–3375. (doi:10.1890/09-1525.1) 21141197

[36] Prévot-Julliard A-C , Pradel R, Julliard R, Grosbois V, Lebreton J-D. 2001 Hatching date influences age at first reproduction in the black-headed gull. Oecologia 127, 62–68. (doi:10.1007/s004420000564) 28547170

[37] Lebreton J-D , Landry P. 1979 Fécondité de la mouette rieuse (Larus ridibundus) dans une colonie importante de la plaine du Forez (Loire, France). Le Gerfaut 69, 159–194.

[38] Lebreton J-D . 1981 Contribution à la dynamique des populations d'oiseaux : modèles mathématiques en temps discret. PhD thesis, Université Claude Bernard, France.

[39] Grosbois V , Tavecchia G. 2003 Modeling dispersal with capture–recapture data: disentangling decisions of leaving and settlement. Ecology 84, 1225–1236. (doi:10.1890/0012-9658(2003)084[1225:MDWCDD]2.0.CO;2)

[40] Grosbois V . 2001 La dispersion : trait d'histoire de vie et paramètre démographique : étude empirique dans une population de mouette rieuse *Larus ridibundus*. PhD thesis, Université de Montpellier, France.

[41] R Core Team . 2022 R: a language and environment for statistical computing Vienna, Austria: R Foundation for Statistical Computing. https://www.R-project.org/.

[42] Brooks ME , Kristensen K, van Benthem KJ, Magnusson A, Berg CW, Nielsen A, Skaug HJ, Mächler M, Bolker BM. 2017 glmmTMB balances speed and flexibility among packages for zero-inflated generalized linear mixed modeling. R J. 9, 378–400. (doi:10.32614/rj-2017-066)

[43] Lenth RV *et al.* 2025 Emmeans: estimated marginal means, aka least-squares means. R package version 1.11.1. https://CRAN.R-project.org/package=emmeans.

[44] Zeileis A , Kleiber C. 2014 Ineq: measuring inequality, concentration, and poverty. R package version 0.2-13. https://CRAN.R-project.org/package=ineq.

[45] Schaub M , Kéry M, Meredith M. 2021 Integrated population models: theory and ecological applications with R and JAGS. London, UK: Academic Press.

[46] Lebreton J-D . 1987 Régulation par le recrutement chez la mouette rieuse Larus ridibundus. Rev. Ecol. Terre Vie 4, 173–182. (doi:10.3406/revec.1987.6267)

[47] Plummer M . 2003 JAGS: a program for analysis of Bayesian graphical models using gibbs sampling. In Proc. 3rd Int. Workshop Distrib. Stat. Comput. Vienna, Austria: Technische Universität Wien.

[48] Kellner K . 2024 JagsUI: a wrapper around ‘rjags’ to streamline ‘JAGS’ analyses. R package version 1.6.2. https://CRAN.R-project.org/package=jagsUI.

[49] Schaub M , Kéry M, Meredith M. 2023 IPMbook: functions and data for the book ‘Integrated population models’. R package version 0.1.5. https://CRAN.R-project.org/package=IPMbook.

[50] Gelman A , Rubin DB. 1992 Inference from iterative simulation using multiple sequences. Stat. Sci. 7, 457–472. (doi:10.1214/ss/1177011136)

[51] Scopel LC , Diamond AW. 2018 Predation and food–weather interactions drive colony collapse in a managed metapopulation of Arctic Terns (*Sterna paradisaea*). Can. J. Zool. 96, 13–22. (doi:10.1139/cjz-2016-0281)

[52] Youngflesh C , Lynch HJ. 2017 Black-swan events: population crashes or temporary emigration? Proc. Natl Acad. Sci. U.S.A. 114, 8953–8954. (doi:10.1073/pnas.1713621114) PMC5664552 29073090

[53] Francesiaz C . 2016 Sélection d'habitat et dynamique spatio-temporelle des colonies chez deux espèces de laridés, la mouette rieuse et le goéland railleur. PhD thesis, Université de Montpellier, France.

[54] Prevot‐Julliard A‐C , Lebreton J‐D. 1999 Spatial organization of foraging within a black-headed gull *Larus ridibundus* colony. IBIS 141, 144–149. (doi:10.1111/j.1474-919X.1999.tb04275.x)

[55] Delgado S , Herrero A, Galarza A, Aldalur A, Zorrozua N, Arizaga J. 2021 Demographic impact of landfill closure on a resident opportunistic gull. Popul. Ecol. 63, 238–246. (doi:10.1002/1438-390X.12083)

[56] Nater CR . 2024 Redefining ‘state-of-the-art’ for integrated population models with immigration. J. Anim. Ecol. 93, 520–524. (doi:10.1111/1365-2656.14087) 38634153

[57] Péron G , Lebreton J-D, Crochet P-A. 2010 Costs and benefits of colony size vary during the breeding cycle in black-headed gulls *Chroicocephalus ridibundus*. J. Ornithol. 151, 881–888. (doi:10.1007/s10336-010-0526-8)

[58] Fortuna MA , Gómez-Rodríguez C, Bascompte J. 2006 Spatial network structure and amphibian persistence in stochastic environments. Proc. R. Soc. B 273, 1429–1434. (doi:10.1098/rspb.2005.3448) PMC1560303 16777733

[59] Oro D , Doak DF, Giménez-Romero Á, Genovart M. 2026 Rare events and their role in ecological dynamics. Trends in Ecology & Evolution. (doi:10.1016/j.tree.2026.07.012) 42637654

[60] Gregory KA , Rumianowski O, Lebreton J-D, Crochet P-A, Besnard A. 2026 Code and data for: change in the demographic dynamics of a spatially structured population of a colonial waterbird after a perturbation affecting a large colony. [Dataset]. Zenodo. (doi:10.5281/zenodo.21995997) PMC13678519 42869470

[61] Gregory KA , Rumianowski O Lebreton J-D Crochet P-A Besnard A. 2026 Supplementary material from: Change in the demographic dynamics of as patially structured population of a colonial waterbird after a perturbation affecting a large colony. FigShare. (doi:10.6084/m9.figshare.c.8693411) PMC13678519 42869470

