# Supplementary material for: Change in the demographic dynamics of a spatially structured population of a colonial waterbird after a perturbation affecting a large colony
Source: Proc Biol Sci. 2026 Oct 14;293(2080):20260388. doi: 10.1098/rspb.2026.0388 (PMC13678519; doi:10.1098/rspb.2026.0388)
Supplement: Supplementary file 1 [file rspb.2026.0388.zip › rspb.2026.0388/rspb20260388_si_001.pdf]

Electronic Supplementary Material

Journal

Proceedings of the Royal Society B: Biological Sciences

Article DOI

10.1098/rspb.2026.0388

Title

Change in the demographic dynamics of a spatially structured population of a colonial waterbird after a perturbation affecting a large colony

Authors

Killian A. Gregory, Odin Rumianowski, Jean-Dominique Lebreton, Pierre-André Crochet & Aurélien Besnard

Keywords

Black-headed gull; *Chroicocephalus ridibundus*; Colonies; Conspecific attraction; Demography; Dispersal; Disturbance; Larids; Patch occupancy; Spatially structured population.

ESM1 – Complements on data collection

Description of the study system

The Forez plain numbers more than 350 ponds, most of them artificial and of strong patrimonial importance (Fig.S1.1). Overall, the surface area of these ponds ranges from less than one hectare to several dozen hectares (BD TOPO®, IGN, 2024) and are generally around one metre deep, except in the ditches and near the sluice gate. These ponds have historically been used for aquaculture (carp, other cyprinids, pike…) in rotation with cereal crop farming (*Document d'objectifs de la zone spéciale de conservation «Etangs du Forez » - site FR8201755*, 2011). Although aquaculture activity has declined, some ponds are still drained in winter to harvest fish, and ponds remain under human management, notably for waterbird hunting. Winter draining has no direct impact on black-headed gulls, since ponds are usually reflooded before the onset of their breeding season, so that the vegetation fringes and islets where gulls nest remain protected from terrestrial predators. Ponds are occasionally kept drained for a full year, however, to promote the mineralisation of mud and organic deposits, control eutrophication, limit cyanobacterial development, and allow occasional maintenance work. These prolonged draining events are the focus of this study, as they overlap with the gulls' breeding season and directly affect breeding site availability. Indeed, colonies do not settle on drained ponds, as they no longer offer protection from terrestrial predators.

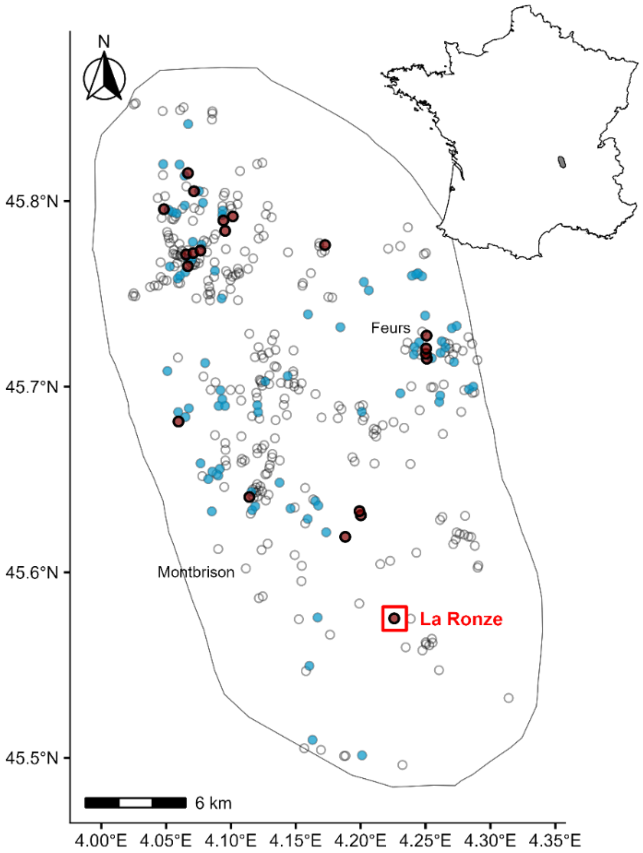

Figure S1.1. Colonies affected by pond draining on the Forez plain. Our study population of black-headed gulls occupies a network of over 350 ponds (circles) located within a lowland agricultural area of about 20x40km² in central France. A hundred of these ponds have been occupied by a colony of gulls for at least one year since the beginning of the monitoring programme in 1976 (blue and red circles). Occasional annual pond draining, carried out as part of their management and historical anthropogenic activities, has prevented some colonies from resettling on their previous breeding site, resulting in local perturbations (red circles). La Ronze pond, which hosted the largest and most stable colony on the plain before it was drained in 2007, is marked with a red square. City names have been displayed as notable landmarks.

Terrestrial counts: from the number of flying adults to the number of breeding pairs (colony size)

The number of breeding pairs is annually estimated based on the number of adults present on the pond occupied by the colony. First, all flying adults in the colony and peripheral roosting sites ("clubs") are counted during a brief take-off induced by experienced observers. This number of flying adults is then converted to the number of breeding pairs in the colony using an empirical correction coefficient of 0.7 (J.-D. Lebreton, *pers. comm.*). This coefficient was determined by comparing the number of nests and diverse estimates of breeding pair numbers, and was progressively validated in the early years of the monitoring programme (see Lebreton, 1981). On average, slightly fewer than 1.5 adults can be observed on the pond per nest: one parent usually stays on the nest, while the other one can either be on the pond next to the nest or in a club – in which case it will also be counted during the induced take-off – or away from the pond to forage. In other words, there are slightly more than 0.66 nests (i.e. breeding pairs) for each observed adult – hence the factor of 0.7.

As this methodology was consistently applied throughout the whole study period, any potential bias in colony size it may have caused should not affect relative variations in colony sizes. The slight imprecision of the empirical coefficient was further accounted for in our study, through the modelling of an observation error for the terrestrial counts.

Corrected colony size for La Ronze colony between 1981 and 2000

Between the 1980s and the 2000s, La Ronze pond hosted a colony numbering over 2000 breeding pairs. Counts of this size were particularly imprecise. In this appendix, we report the methodology detailed in Grosbois (2001) to obtain more precise colony size estimates.

Every year between 1981 and 2000, observers at La Ronze (LR) colony recorded the number of ringed and unringed individuals during ring-reading sessions. We refer to such an event, where data is collected on the number of ringed and unringed individuals, as a 'passage'.

Let $P_{O/1P}$ be the probability that an individual (ringed or not) is observed during one passage. We assume the individuals can only be seen once during each passage, given they spend the majority of their time in the colony in close vicinity to their nests. Considering two passages per year, the number of observations N_O is obtained by summing contributions across three possible detection histories:

- An individual seen during both passages contributes 2 observations, with probability ${P_{O/1P}}^2$
- An individual seen during the first passage only contributes 1 observation, with probability $P_{O/1P}(1 - P_{O/1P})$
- An individual seen during the second passage only contributes 1 observation, with probability $(1 - P_{O/1P})P_{O/1P}$

The expected total number of observations $E(N_O)$ is therefore related to the total size of the colony N_N in a given year, which we are aiming for:

$$E(N_O) = N_N \cdot (2{P_{O/1P}}^2 + 2P_{O/1P}(1 - P_{O/1P})) \qquad (1)$$

By simplifying equation (1), we obtain $E(N_O) = N_N \cdot 2P_{O/1P}$, which yields the following estimator $\widehat{N}_N$ for colony size in a given year:

$$\widehat{N}_N = \frac{N_O}{2\widehat{P}_{O/1P}} \qquad (2)$$

Estimating N_N therefore requires estimating $P_{O/1P}$, which can be obtained by distinguishing observations of ringed and unringed individuals. In the sampling design described here, the probability $\widehat{P}_{CR}$ that a ringed bird is resighted on a colony can be defined as the probability of being observed and identified in at least one of the two passages, requiring both observation (with probability $\widehat{P}_{O/1P}$ assuming ringed and unringed individuals have equal detection probability) and successful ring identification given the individual is ringed and observed (with probability $\widehat{P}_{C/O}$):

$$\widehat{P}_{CR} = 1 - (1 - \widehat{P}_{O/1P}\widehat{P}_{C/O})^2$$

From this equation, we get:

$$\widehat{P}_{O/1P} = \frac{1 - \sqrt{1 - \widehat{P}_{CR}}}{\widehat{P}_{C/O}} \qquad (3)$$

Combining equations (2) and (3) yields the final estimator for colony size:

$$\hat{N}_N = \frac{N_O}{2\left[\frac{1-\sqrt{1-\hat{P}_{CR}}}{\hat{P}_{C/O}}\right]} \qquad (4)$$

In equation (4), N_O is recorded in the field. $\hat{P}_{C/O}$ is estimated from the number of resightings where the observed ringed individual could be identified, N_C, and the number of resightings where the observed ringed individual could not be identified, $N_{\bar{C}}$:

$$\hat{P}_{C/O} = \frac{N_C}{N_C + N_{\bar{C}}} \qquad (5)$$

Recapture probability $\hat{P}_{CR}$ can be estimated from capture-recapture data on adults breeding in the colony. For LR colony, this probability was estimated using a single-site capture-recapture model with constant survival and time-varying recapture probability. This model structure was selected through a model selection procedure based on AICc (Grosbois 2001).

In addition, an expression for the variance of $\hat{N}_N$, $\sigma^2(\hat{N}_N)$, was derived using the delta method (Rao, 1973). Without expressing the term related to the covariance of $\hat{P}_{CR}$ and $\hat{P}_{C/O}$:

$$\sigma^2(\hat{N}_N) \approx \left[\frac{N_O}{2(1-\sqrt{1-\hat{P}_{CR}})}\right]^2 \sigma^2(\hat{P}_{C/O}) + \left[\frac{N_O\hat{P}_{C/O}}{4[(1-\sqrt{1-\hat{P}_{CR}})^2\sqrt{1-\hat{P}_{CR}}]}\right]^2 \sigma^2(\hat{P}_{CR}) \qquad (6)$$

In equation (6), $\sigma^2(\hat{P}_{C/O}) = \frac{\hat{P}_{C/O}(1-\hat{P}_{C/O})}{N_C+N_{\bar{C}}}$ and $\sigma^2(\hat{P}_{CR})$ is obtained from the capture-recapture model.

Data and estimates reported by Grosbois (2001) are given in table S1.1. The number of breeding pairs used in this article for LR colony between 1981 and 2000 correspond to half the total number of breeders reported by Grosbois (2001), assuming a balanced sex ratio.

Table S1.1. Estimation of the number of breeders on La Ronze colony between 1981 and 2000 based on the observation of ringed and not ringed individuals, reported by Grosbois (2001). Column names refer to parameters presented above.

**Year**	N_O	$\hat{P}_{C/O}$	$\sigma^2(\hat{P}_{C/O})$	$\hat{P}_{CR}$	$\sigma^2(\hat{P}_{CR})$	$\hat{N}_N$	$\sigma^2(\hat{N}_N)$
**1981**	1002	0.94	0.0004	0.20	0.0043	**4370**	2261561
**1982**	818	0.69	0.0022	0.17	0.0015	**3168**	612770
**1983**	1005	0.78	0.0015	0.12	0.0010	**6566**	3356844
**1984**	1453	0.76	0.0011	0.16	0.0010	**6665**	2052900
**1985**	1506	0.71	0.0010	0.19	0.0010	**5431**	955803
**1986**	1420	0.71	0.0010	0.14	0.0006	**7145**	1961577
**1987**	1519	0.82	0.0008	0.15	0.0006	**8059**	1842307
**1988**	1719	0.80	0.0007	0.23	0.0007	**5585**	519864
**1989**	2740	0.69	0.0006	0.22	0.0007	**7893**	1029634
**1990**	1674	0.81	0.0007	0.15	0.0004	**8487**	1505526
**1991**	1255	0.89	0.0008	0.10	0.0003	**10428**	3180979
**1992**	1495	0.70	0.0010	0.09	0.0003	**10877**	4306477
**1993**	1734	0.68	0.0009	0.15	0.0005	**7310**	1238054
**1994**	1289	0.79	0.0009	0.17	0.0005	**5882**	771546
**1995**	1082	0.78	0.0011	0.13	0.0005	**6027**	1074419
**1996**	1210	0.77	0.0011	0.14	0.0005	**6584**	1412298
**1997**	1376	0.75	0.0011	0.12	0.0005	**8499**	2876125
**1998**	1443	0.77	0.0010	0.12	0.0005	**9225**	3376687
**1999**	1178	0.73	0.0013	0.13	0.0005	**6639**	1637026
**2000**	882	0.65	0.0017	0.08	0.0004	**7000**	2980960

References

Grosbois V. 2001 La dispersion : trait d'histoire de vie et paramètre démographique : étude empirique dans une population de mouette rieuse *Larus ridibundus*. Université de Montpellier 2.

Lebreton J-D. 1981 Contribution à la dynamique des populations d'oiseaux : modèles mathématiques en temps discret. Université Claude Bernard.

Summary of the data used in the analysis

Table S1.2. Summary of the data used in the analysis. Coloured cells indicate years and ponds where colonies were present (green for colonies with terrestrial counts, blue for colonies with corrected counts obtained using the protocol described in this Appendix), or years in which ponds were drained (red). White cells denote years where colonies were not present on the ponds, not missing data. Letters indicate whether individuals were ringed (R) or observed (O) as part of the capture-recapture monitoring. Depending on data availability within each period on which the Integrated Population Model (IPM) was run, this capture-recapture data was either used (black) or not (white).

	1976	1977	1978	1979	1980	1981	1982	1983	1984	1985	1986	1987	1988	1989	1990	1991	1992	1993	1994	1995	1996	1997	1998	1999	2000
**La Ronze (LR)**	R	R	RO	RO	RO	RO	RO	RO	RO	RO	RO	RO	RO	RO	RO	RO	RO	RO	RO	RO	RO	RO	RO	RO	RO
**Les Marquants (MA)**										R	R		R	R				R	RO	RO	RO	RO	O	RO	RO
**La Verchère (VE)**				R	R	R		R	R	R	R	R			R	R	R			RO	RO		RO	RO	RO
**La Vallon 5 (V5)**																					RO	RO	RO	RO	
**La Vergnat Sud (VS)**																									
**Les Champs Blancs (LA)**																									
**Sury (SV)**				R	R	R																R			
**Vieux Ouest (WV)**	R	R																		R					
**La Sauzée (SZ)**																									
**Other colonies**	R	R	R	R	RO	RO	RO	RO	RO	RO	RO	RO	RO	RO	RO	R	R	RO	RO	RO	RO	RO	RO	RO	RO
**Data on drained ponds**																									
**Periods for the IPM**	-	-	-	-	-	-	-	-	-	-	Period 1														

	2001	2002	2003	2004	2005	2006	2007	2008	2009	2010	2011	2012	2013	2014	2015	2016	2017	2018	2019	2020	2021	2022	2023	2024	2025
**La Ronze (LR)**	RO	RO	RO	RO	RO	RO		RO	RO	RO	RO	RO	RO	RO	RO	RO	RO	RO	RO	O	R	R	R	RO	R
**Les Marquants (MA)**	RO		RO	RO	RO	RO	RO	RO			RO		RO	O				RO	O						
**La Verchère (VE)**	RO	RO	RO			RO	O																		
**La Vallon 5 (V5)**																									
**La Vergnat Sud (VS)**						O	O	O							RO	RO	RO	RO							
**Les Champs Blancs (LA)**							O	RO								RO	RO								
**Sury (SV)**							RO	RO	RO		RO	O	RO		RO	O	RO								
**Vieux Ouest (WV)**							RO	RO	RO	RO	RO	RO	RO	RO		RO	RO	O							
**La Sauzée (SZ)**											RO	RO				O									
**Other colonies**	R	O	R	R	RO				RO						RO	RO	RO	RO							
**Data on drained ponds**																									
**Periods for the IPM**						Period 2	Period 3				Period 4									-	-	-	-	-	-

Legend:

- Colony present (terrestrial counts)
- Colony present (corrected counts)
- Colony absent
- Drained pond

- R — Ringing
- O — Observations of ringed individuals
- **R/O** — Ringing data not used in the IPMs
