# Supplementary material for: Change in the demographic dynamics of a spatially structured population of a colonial waterbird after a perturbation affecting a large colony
Source: Proc Biol Sci. 2026 Oct 14;293(2080):20260388. doi: 10.1098/rspb.2026.0388 (PMC13678519; doi:10.1098/rspb.2026.0388)
Supplement: Supplementary file 1 [file rspb.2026.0388.zip › rspb.2026.0388/rspb20260388_si_002.pdf]

Electronic Supplementary Material

Journal

Proceedings of the Royal Society B: Biological Sciences

Article DOI

10.1098/rspb.2026.0388

Title

Change in the demographic dynamics of a spatially structured population of a colonial waterbird after a perturbation affecting a large colony

Authors

Killian A. Gregory, Odin Rumianowski, Jean-Dominique Lebreton, Pierre-André Crochet & Aurélien Besnard

Keywords

Black-headed gull; *Chroicocephalus ridibundus*; Colonies; Conspecific attraction; Demography; Dispersal; Disturbance; Larids; Patch occupancy; Spatially structured population.

ESM2 – Integrated Population Model

Life-cycle formalisation

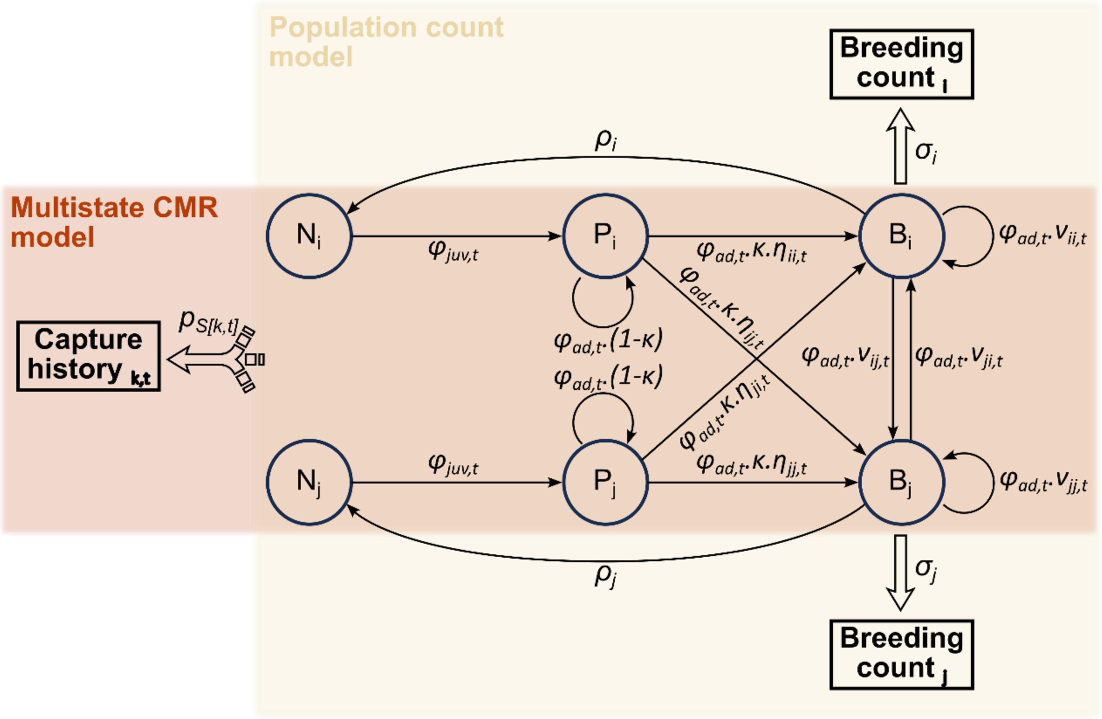

Figure S2.1. Life cycle underlying the integrated population model. Nodes refer to the three breeding stages of the population: nestlings (N), pre-breeders (P) and breeders (B). Stages are defined for each monitored pond, although only two are displayed for clarity (i, j). They represent the different compartements of the population, and therefore the different states s in which an individual k can be resighted in year t. Arrows show the transitions between states. Parameters are involved in either or both the population count model (beige box) and the multistate capture-mark-recapture model (red box): survival (φ, juveniles and adults), fecundity (ρ), recruitement (κ), natal dispersal (η), breeding dispersal (v), individual recapture probability (p), observation error for population counts (σ).

Prior distribution of parameters

Mean survival probabilities ($\overline{\phi_{ad}}$, $\overline{\phi_{Juv}}$) and mean detection probabilities ($\overline{p_l}$) had uniform priors between 0 and 1. Standard deviations of random year effects on the *logit* scale followed uniform priors between 0.0001 and 1 for survival, and between 0.01 and 100 for detection.

Fecundity ρ_i had a uniform prior restricted between 0 and 1, corresponding to up to one female offspring per breeding pair on average, assuming a maximum of two fledglings per pair in a scenario of balanced sex ratio (Eq.1) (Lebreton 1987).

Natal and breeding dispersal probabilities followed Dirichlet(1) distributions, ensuring values between 0 and 1, and that dispersal probabilities from a given colony summed to one.

Observation error proportionality factors $\sigma_{LR,SAT}$ for population counts $C_{i,t}$ had uniform priors between 0 and 0.30 for LR, and between 0 and 0.15 for satellite colonies. This reflects the additional uncertainty in the counts for LR colony, which reached particularly large breeding numbers and was subject to various counting methods depending on the period.

Initial breeder numbers had uniform priors with bounds set at $\pm$80% of observed counts. Initial pre-breeder numbers had uniform priors with bounds set at $\pm$80% of numbers calculated using observed breeder counts and the proportion of pre-breeders predicted from the stable stage distribution of a matrix population model (Schaub and Kéry 2021), which was constructed using estimates from a previous study (Péron et al. 2010).

Data simulation

The performance of the integrated population model was assessed on simulated data. We simulated population counts and capture-recapture data for five colonies (i) over 20 years (t). The five colonies included one large colony with characteristics similar to those of la Ronze colony (large and attractive for breeding dispersers), three satellite colonies with a smaller number of breeders, and one colony with no ringing effort to mimic the "Alive Elsewhere" state. With three stages (nestlings, pre-breeders and breeders), this corresponded to 15 capture states plus an additional state for individuals that were demographically dead.

Constants and parameter values for the simulations are summarised in Table S2. Values for the demographic parameters were chosen based on the estimates from a previous study of the same population (Péron et al. 2010). Similar to the model structure, annual survival probabilities for adults $\phi_{ad,t}$ and juveniles $\phi_{juv,t}$ were simulated using mean interannual survival probabilities ($\overline{\phi_{ad}}, \overline{\phi_{Juv}}$) and a normal random effect modelled on the *logit* scale using age-specific standard deviations ($\sigma_{ad}^{(\phi)}$, $\sigma_{juv}^{(\phi)}$). This generated annual survival probabilities ranging from 0.74 to 0.94 for adults, and from 0.10 to 0.27 for juveniles. The same approach was used to simulate annual resighting probabilities $p_{i,t}$ for each colony, based on mean detection probabilities ($\overline{p_\iota}$) and a normal random effect modelled on the *logit* scale ($\sigma_i^{(p)}$). This generated annual detection probabilities ranging from 0.02 to 0.68, consistent with the heterogeneity of resighting probabilities in our study population (Prévot-Julliard 1996). To simulate years with no reading effort, the detection probability was set to zero for randomly selected colony-years using a Bernoulli distribution with a probability of 0.1. This corresponded to around ten percent of the colony-years over the monitoring period.

Natal and breeding dispersal probabilities were chosen to reflect a diversity of dispersal patterns between colonies, while remaining consistent with the estimates of Péron et al. (2010). Each year, a random number of extinct colonies were drawn among the three satellite colonies using a Poisson distribution with parameter 0.4. This resulted in satellite colonies becoming extinct for one to three years during the monitoring period. As in the model, extinction was then modelled by setting the dispersal probabilities towards the extinct colonies to 0 in the year of extinction, while normalising the remaining dispersal probabilities so that they summed to one for each departure colony.

The initial number of breeders was similar to that in the real dataset in 1986. The number of ringed nestlings and breeders in each year was similar to that in the real dataset between 1986 and 2005. These demographic parameters were used to calculate the initial proportion of pre-breeders in each colony, based on the predicted age structure from a Leslie matrix. As described by eq. 3-4 (see main document), observation error for breeding counts was modelled as a normal random effect proportional to colony size, with a proportionality factor $\sigma_{LR,SAT}$ that differed between the large colony and the others.

Based on these parameters, count data was simulated using eq.1-4, and capture-data was simulated using Ψ_t and P_t matrices (see main document).

All simulated parameter values fell within the posterior distributions of the model estimates (Fig.S2.2-5. The difference between the simulated values and the posterior means was highest for natal dispersal probabilities. However, this was to be expected given the low number of data points for these unique events in an individual's life, which makes natal dispersal a difficult parameter to estimate.

Table S2. Choice of parameters for data simulation.

Constants and sample sizes	
Number of colonies i (or j)	5 colonies:<br>- 1 large colony<br>- 3 satellite colonies<br>- 1 "Alive Elsewhere", grouping all unmonitored colonies
Number of years of monitoring t	20 years
Number of life stages	3 (nestlings, pre-breeders, breeders)
Number of capture states s	5 colonies $\times$ 3 life stages $+$ 1 "dead" state $=$ 16 capture states
Population counts – Initial number of females $B_{i,1}$	Large colony: 3800 females<br>Satellite colonies: 1000, 500, 10 females respectively<br>Alive elsewhere: 3600 females
Population counts – Initial proportion of prebreeders $P_{i,1}$	Large colony: 15 %<br>Satellite colonies: 6 %<br>Alive elsewhere: 6 %
Observation error for population counts – Proportionality factor for normal random effect $\sigma_{LR,SAT}$	Large colony: 0.20<br>Satellite colonies: 0.10<br>Alive elsewhere: 0.10
**Demographic parameters**	
Mean apparent adult survival probability $\overline{\phi_{ad}}$	0.85
Mean apparent juvenile survival probability $\overline{\phi_{juv}}$	0.20
Standard deviation on the *logit* scale for adult survival random effect $\sigma_{ad}^{(\phi)}$	0.30
Standard deviation on the *logit* scale for juvenile survival random effect $\sigma_{juv}^{(\phi)}$	0.50
Fecundity (females with balanced sex-ratio) ρ_i	Large colony: 1.7/2 = 0.85<br>Satellite colonies: 0.6/2 = 0.3<br>Alive elsewhere: 0.6/2 = 0.3
Recruitment probability κ	0 for first year individuals; 1 for individuals aged 2 or more
Natal dispersal probabilities between colonies $\eta_{j,i,t}$	(see matrix below)
Breeding dispersal probabilities between colonies $\nu_{j,i,t}$	(see matrix below)
Mean detection probability on colony i $\overline{p_i}$	$Uniform(min\ =\ 0.05, max\ =\ 0.20)$
Standard deviation on the *logit* scale for detection random effect $\sigma_i^{(p)}$	$Uniform(min\ =\ 0.50, max\ =\ 1.00)$

Natal dispersal probabilities between colonies $\eta_{j,i,t}$

From\To	Large colony	Satellite 1	Satellite 2	Satellite 3	Alive Elsewhere
Large colony	0.40	0.30	0.20	0	0.10
Satellite 1	0	0.50	0.20	0.20	0.10
Satellite 2	0	0.20	0.70	0	0.10
Satellite 3	0	0.10	0.10	0.80	0
Alive Elsewhere	0	0.30	0.20	0.20	0.30

Breeding dispersal probabilities between colonies $\nu_{j,i,t}$

From\To	Large colony	Satellite 1	Satellite 2	Satellite 3	Alive Elsewhere
Large colony	1	0	0	0	0
Satellite 1	0.10	0.60	0.10	0	0.20
Satellite 2	0.20	0.30	0.50	0	0
Satellite 3	0.10	0.20	0.20	0.40	0.10
Alive Elsewhere	0.015	0.015	0.015	0.015	0.94

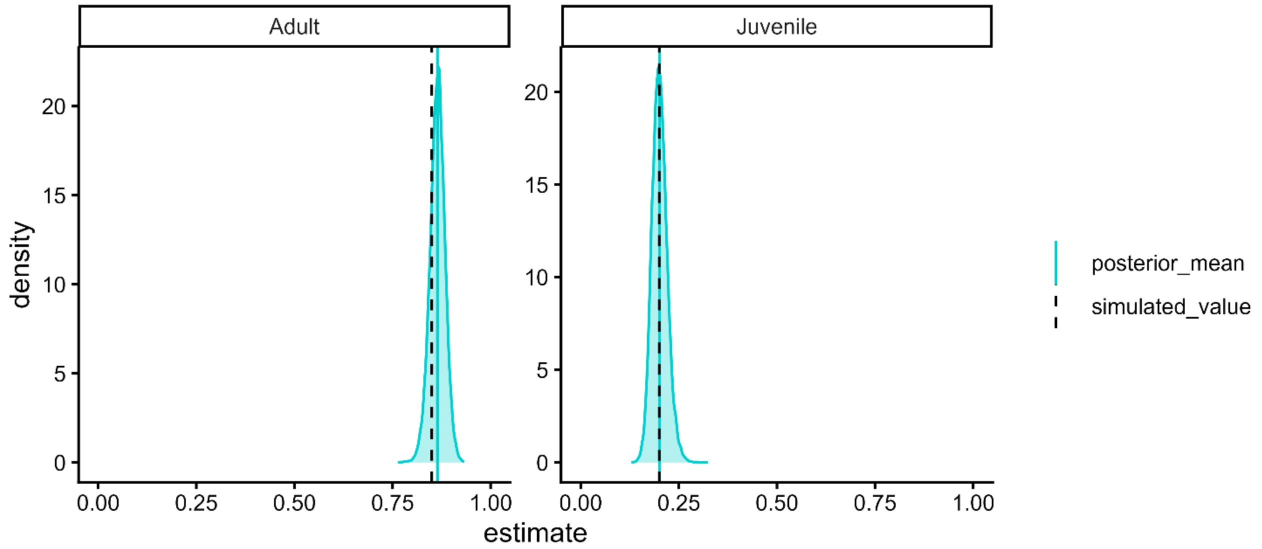

Figure S2.2. Comparison of the simulated and estimated values for mean survival probabilities. The black dashed lines show the true simulated value for the parameters. The blue distributions show the posterior distribution of the estimates for one run of the integrated population model. The blue vertical lines show the mean of these distributions.

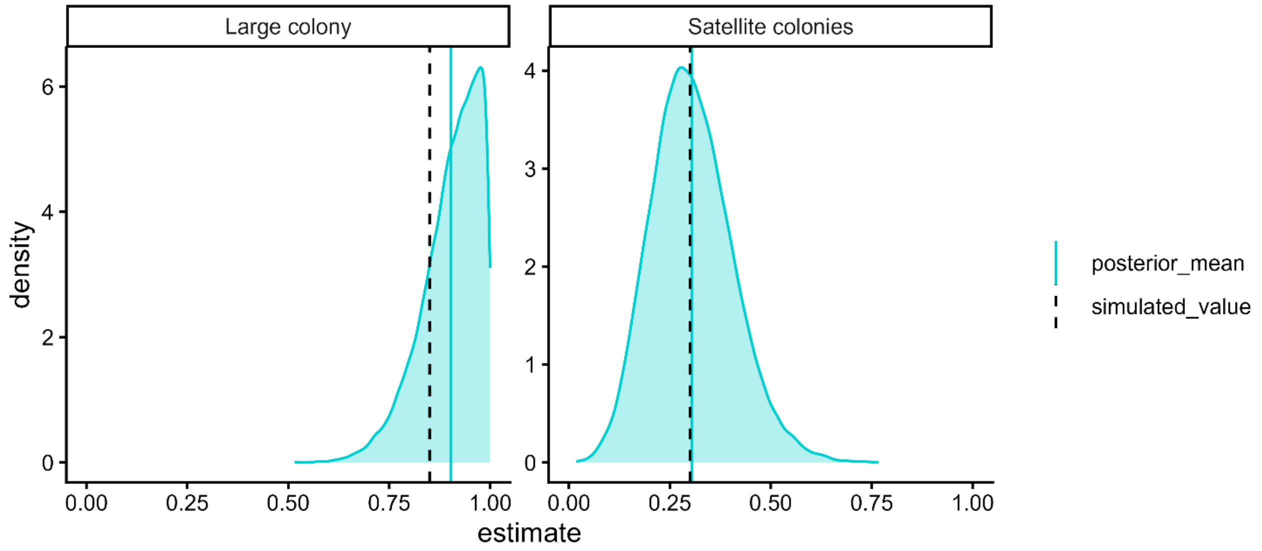

Figure S2.3. Comparison of the simulated and estimated values for fecundity. The black dashed lines show the true simulated value for the parameters. The blue distributions show the posterior distribution of the estimates for one run of the integrated population model. The blue vertical lines show the mean of these distributions.

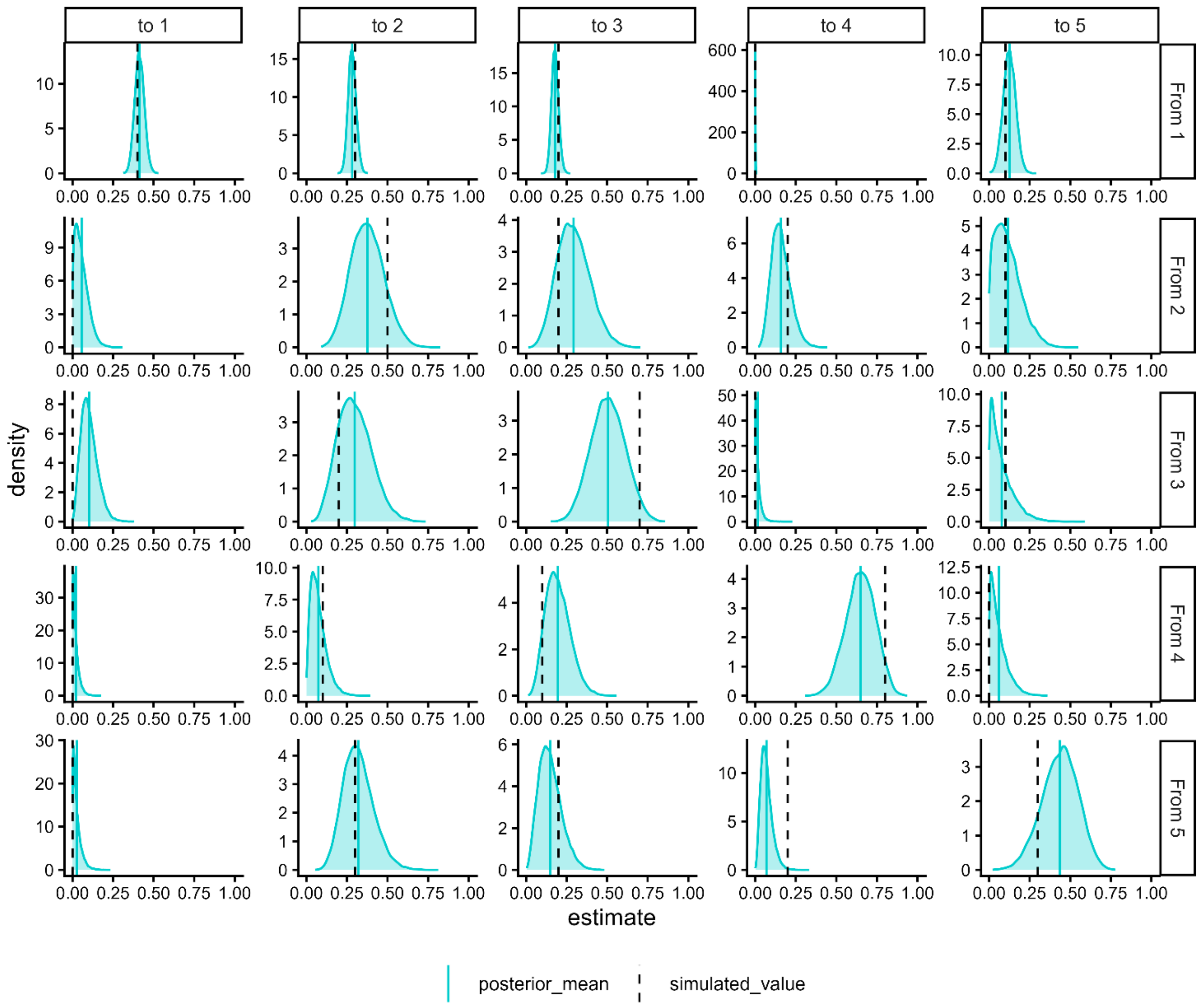

Figure S2.4. Comparison of the simulated and estimated values for natal dispersal. Rows correspond to departure colonies occupied in year t and columns correspond to destination colonies occupied in year $(t + 1)$. The black dashed lines show the true simulated value for the parameters. The blue distributions show the posterior distribution of the estimates for one run of the integrated population model. The blue vertical lines show the mean of these distributions.

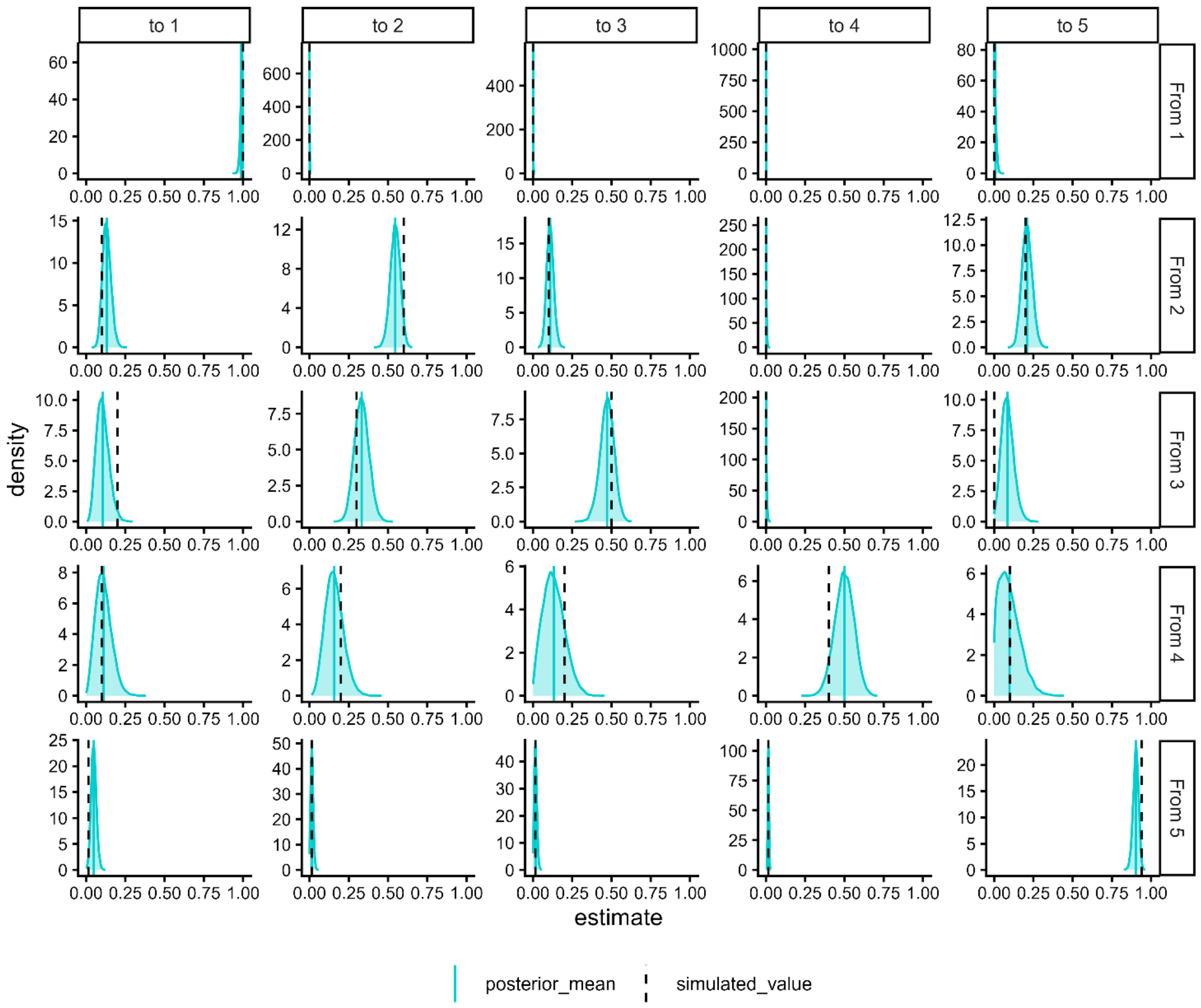

Figure S2.5. Comparison of the simulated and estimated values for breeding dispersal. Rows correspond to departure colonies occupied in year t and columns correspond to destination colonies occupied in year $(t + 1)$. The black dashed lines show the true simulated value for the parameters. The blue distributions show the posterior distribution of the estimates for one run of the integrated population model. The blue vertical lines show the mean of these distributions.

References

Lebreton J-D. 1987 Régulation par le recrutement chez la mouette rieuse *Larus ridibundus*. *Revue d'Ecologie, Terre et Vie* **Suppl. 4**, 173–182.

Péron G, Crochet P-A, Doherty Jr. PF, Lebreton J-D. 2010 Studying dispersal at the landscape scale: efficient combination of population surveys and capture–recapture data. *Ecology* **91**, 3365–3375. (doi:10.1890/09-1525.1)

Prévot-Julliard A-C. 1996 Rôle de la dispersion dans la dynamique d'une population de mouette rieuse "*Larus ridibundus L.*". Université de Montpellier 2.

Schaub M, Kéry M. 2021 *Integrated Population Models: Theory and Ecological Applications with R and JAGS*. London: Academic Press. (doi:10.1016/C2019-0-02015-8)
