# Supplementary material for: Change in the demographic dynamics of a spatially structured population of a colonial waterbird after a perturbation affecting a large colony
Source: Proc Biol Sci. 2026 Oct 14;293(2080):20260388. doi: 10.1098/rspb.2026.0388 (PMC13678519; doi:10.1098/rspb.2026.0388)
Supplement: Supplementary file 1 [file rspb.2026.0388.zip › rspb.2026.0388/rspb20260388_si_003.pdf]

Electronic Supplementary Material

Journal

Proceedings of the Royal Society B: Biological Sciences

Article DOI

10.1098/rspb.2026.0388

Title

Change in the demographic dynamics of a spatially structured population of a colonial waterbird after a perturbation affecting a large colony

Authors

Killian A. Gregory, Odin Rumianowski, Jean-Dominique Lebreton, Pierre-André Crochet & Aurélien Besnard

Keywords

Black-headed gull; *Chroicocephalus ridibundus*; Colonies; Conspecific attraction; Demography; Dispersal; Disturbance; Larids; Patch occupancy; Spatially structured population.

ESM3 – Complementary results

Consequences of pond draining on patch dynamics

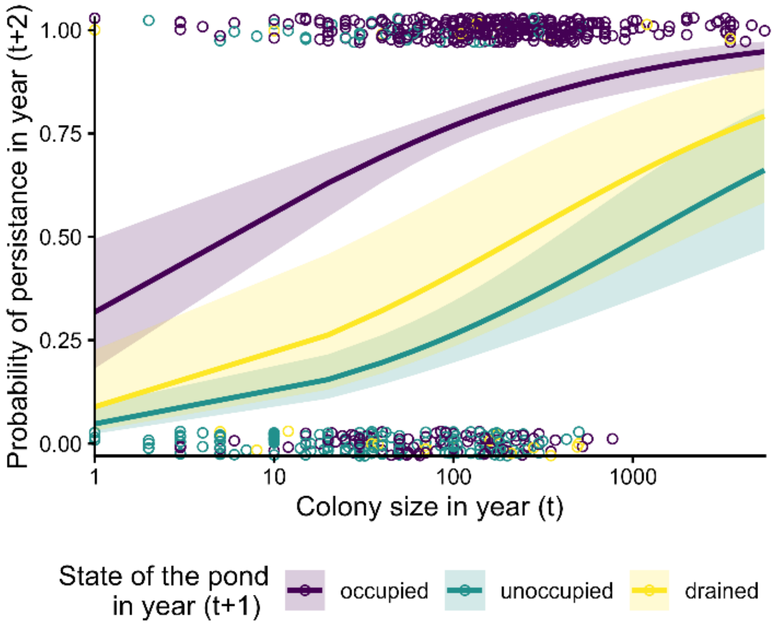

Figure S3.1. Persistence probability in year (t+2) for a colony present in year (t) depending on its size and on the state of the pond in year (t+1). Data points displayed with empty circles and a vertical jitter correspond to persistence/recolonisation (1) or extinction events (0) in year t+2 for colonies active in year t. Lines and shaded areas show the mean and 95% confidence interval of the predictions of the binomial generalised linear mixed model that relates occupancy probability in year t+2 and colony size in year t, when the pond was occupied (purple), unoccupied (green) or drained (yellow) in year t+1.

Heterogeneity in colony size across the study period

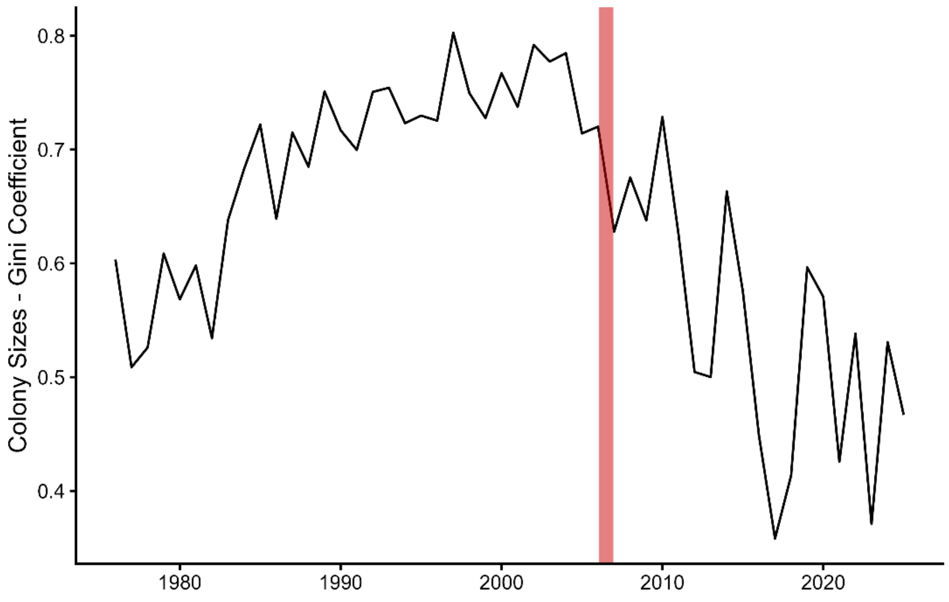

Figure S3.2. Heterogeneity in colony sizes from 1976 to 2025. Heterogeneity was quantified using Gini coefficients, which were calculated based on the number of breeders on all occupied ponds within the population each year during the monitoring period. Higher Gini coefficients indicate greater heterogeneity in colony sizes. The vertical band marks the perturbation that occurred at La Ronze pond in 2007. The initial increase in heterogeneity at the start of the monitoring period coincided with an increase in the total population size and an accumulation of breeders at La Ronze colony.

Demographic estimates of the integrated models

Table S3. Posterior mean and 95% credible interval of some demographic parameters estimated by the integrated population models. Results are shown for three periods of the four periods: before the perturbation (1986-2006), shortly after the perturbation (2007-2010), and a few years after the perturbation (2010-2019).

	1986-2006		2007-2010		2010-2019	
	Est.	CI (95%)	Est.	CI (95%)	Est.	CI (95%)
**Mean apparent adult survival**	0.84	[0.79,0.88]	0.75	[0.56,0.90]	0.87	[0.78,0.93]
**Standard deviation of apparent adult survival**	0.67	[0.39,0.96]	0.83	[0.51,0.99]	0.89	[0.67,1.00]
**Mean apparent juvenile survival**	0.17	[0.14,0.22]	0.47	[0.21,0.80]	0.34	[0.26,0.42]
**Standard deviation of apparent juvenile survival**	0.56	[0.38,0.82]	0.55	[0.04,0.98]	0.33	[0.03,0.79]
**Fecundity in la Ronze colony**	0.97	[0.91,1.00]	0.07	[0.00,0.57]	0.24	[0.00,0.95]
**Fecundity in satellite colonies**	0.97	[0.91,1.00]	0.00	[0.00,0.01]	0.61	[0.19,0.90]
**Breeding fidelity in la Ronze colony**	0.98	[0.97,0.99]	0.68	[0.49,0.90]	0.58	[0.50,0.65]
**Breeding fidelity in les Marquants (satellite colony)**	0.35	[0.26,0.44]	0.23	[0.08,0.45]	0.10	[0.03,0.21]
**Natal fidelity in la Ronze colony**	0.78	[0.73,0.83]	0.23	[0.05,0.55]	0.25	[0.15,0.37]
**Natal fidelity in les Marquants (satellite colony)**	0.36	[0.24,0.51]	0.19	[0.01,0.52]	0.19	[0.03,0.47]

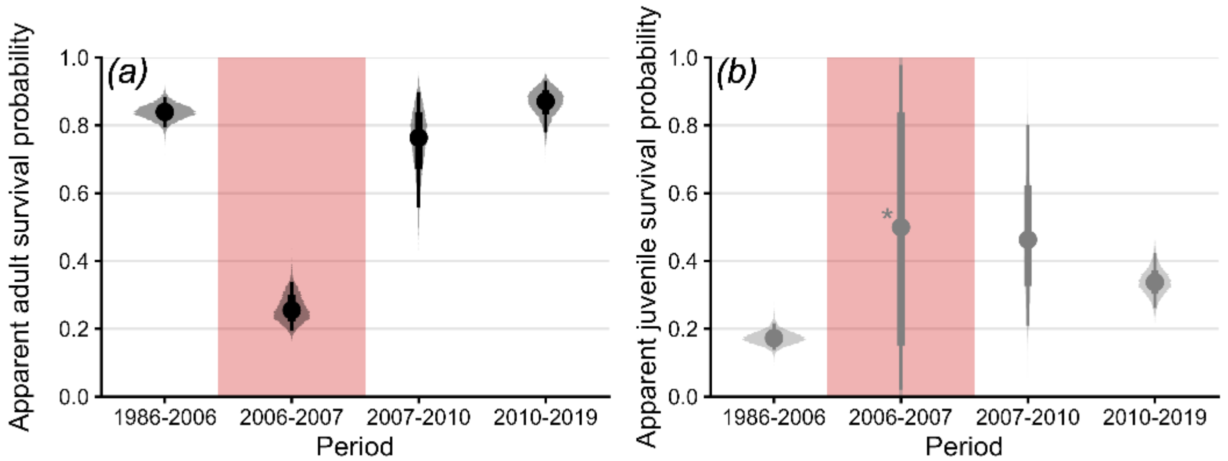

Figure S3.3. Variation in apparent survival of the black-headed gulls over the study period. The vertical band marks the perturbation that occurred at La Ronze (LR) in 2007. (a) Posterior distribution of mean apparent survival probability for adults in the SSP. (b) Posterior distribution of mean apparent survival probability for juveniles in the SSP. Values for adults and pre-breeders are in dark and values for juveniles are in grey. The grey asterisk marks the estimate of juvenile survival in 2007, which was non identifiable by the model.

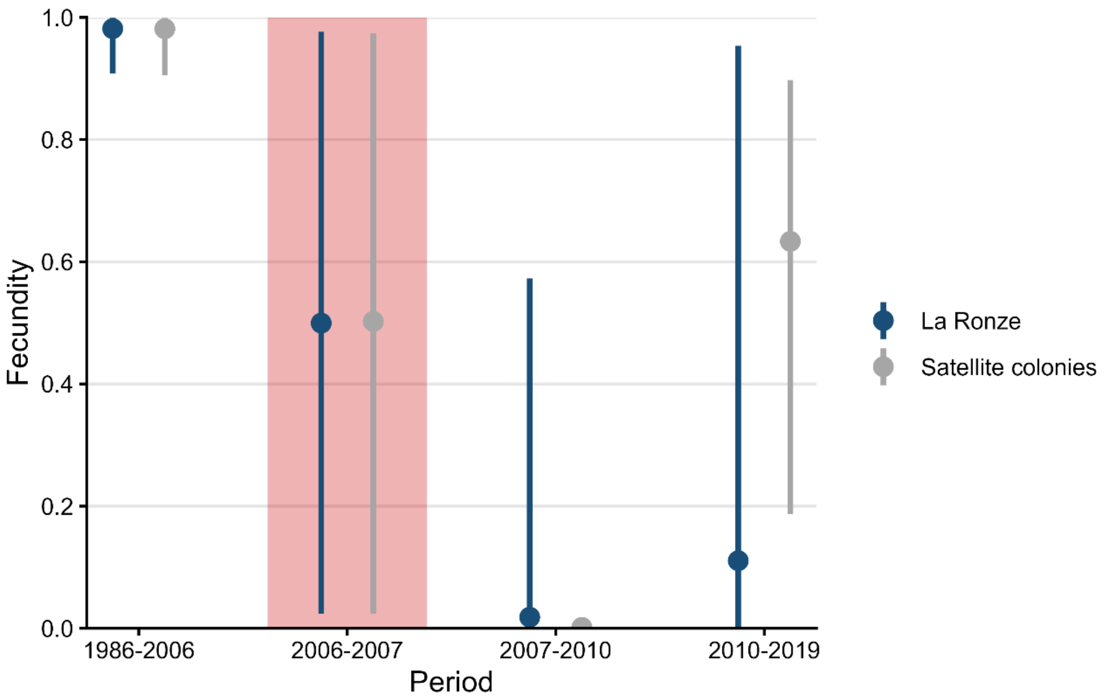

Figure S3.4. Fecundity estimates of the integrated population models from 1986 to 2019. Median and 95% credible interval are displayed for la Ronze (blue) and satellite colonies (grey). The vertical band marks the perturbation that occurred at La Ronze (LR) in 2007.

Correlation between dispersal parameters and colony size

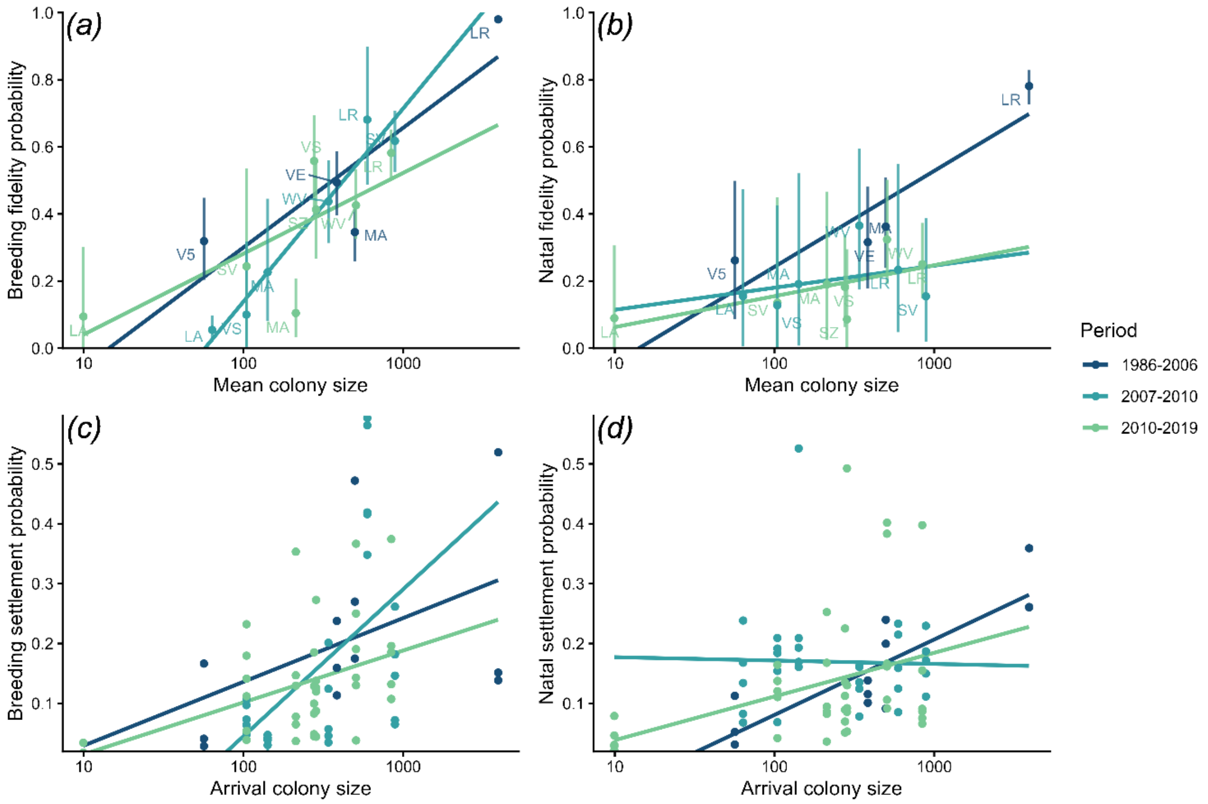

Figure S3.5. Correlation between dispersal parameters and colony size before and after the perturbation. Mean fidelity for breeding dispersal (a) and natal dispersal (b) in relation to colony size. Mean settlement probability for breeding dispersal (c) and natal dispersal (d), given that individuals do not remain on the same site, in relation to the size of the destination colony. 95% credibility intervals are only displayed for fidelity (a, b) to avoid overcharging the plots. Colony sizes have been log-transformed. Results are shown for three of the study-periods: before the perturbation (1986-2006, dark blue), just after the perturbation (2007-2010, light blue) and in the mid-term after the perturbation (2010-2019, green). Although Spearman tests were used to determine the correlation between dispersal parameters and colony size, linear regressions are displayed for visualisation purposes.

Variation in colony sizes across the study period

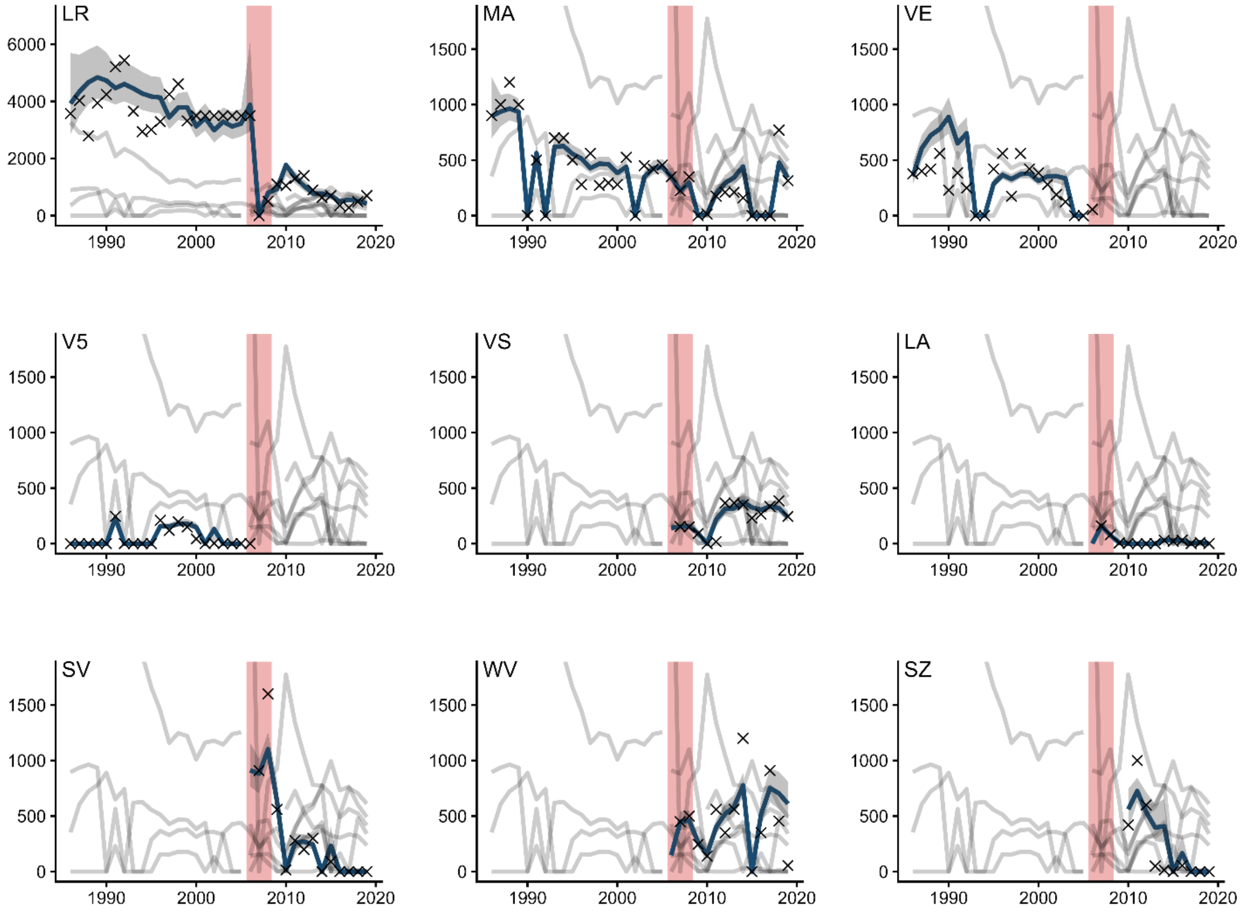

Figure S3.6. Estimated number of breeding pairs on the colonies monitored by capture-mark-recapture from 1986 to 2019. Each panel corresponds to a colony for the period during which it was being monitored: La Ronze (LR), Les Marquants (MA), La Verchère (VE), La Vallon 5 (V5), La Vergnat Sud (VS), Les Champs Blancs (LA), Sury (SV), Vieux Ouest (WV), La Sauzée (SZ). Black crosses correspond to raw count data. Black lines and shaded areas represent the estimated means and 95% credible intervals from the integrated population models for the number of breeders in each colony. Estimates for the other colonies are displayed in grey in each panel for comparison purposes. The red vertical band highlights the draining of LR in 2007.

Proportion of pre-breeders across the study period

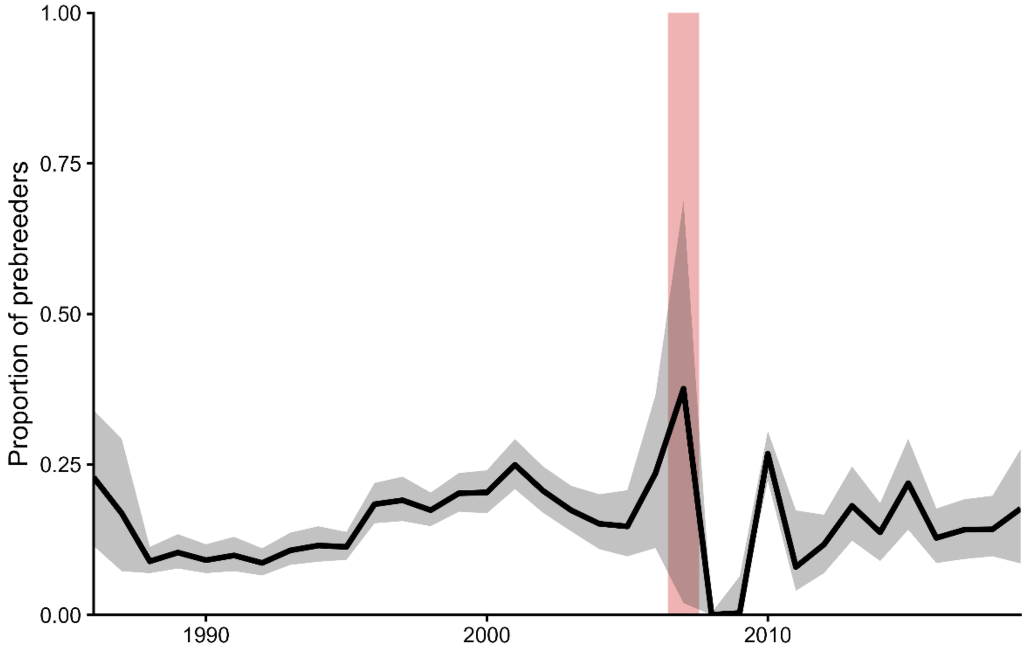

Figure S3.7. Estimated proportion of pre-breeders in the population from 1986 to 2019. Black lines and shaded areas represent the estimated means and 95% credible intervals from the integrated population models. The red vertical band highlights the draining of LR in 2007.
